# Characteristics and driving factors of power generation performance in microbial fuel cells: an analysis based on the CNKI database

**DOI:** 10.3389/fmicb.2025.1620539

**Published:** 2025-06-13

**Authors:** Ruikai Zhang, Wentao Dai, Hongyong Xiang, Jie Chen, Ting Yi, Jiayi Li, Jiebin Zhang, Qiuxi Yang, Rui Xiao, Xiang Li

**Affiliations:** ^1^School of Hydraulic and Ocean Engineering, Changsha University of Science and Technology, Changsha, China; ^2^Key Laboratory of Dongting Lake Aquatic Eco-Environmental Control and Restoration of Hunan Province, Changsha University of Science and Technology, Changsha, China; ^3^Hubei Provincial Key Laboratory of Basin Water Resource and Eco-Environmental Sciences, Changjiang River Scientific Research Institute, Wuhan, China; ^4^Engineering and Technical Center of Hunan Provincial Environmental Protection for River-Lake Dredging Pollution Control, Changsha, China

**Keywords:** electricity performance, MFC, power density, current density, coulombic efficiency

## Abstract

Microbial fuel cells (MFCs) have become one of the most promising technologies in the field of ecology and environmental science due to their dual functions of power generation and pollutant removal. However, the generally low power generation performance of MFCs is one of the bottlenecks constraining their development, and numerous studies have focused on the improvement of power generation performance. The majority of previous empirical studies were based on single experimental data, which means there may be large differences in experimental conditions and settings, leading to various or even contradictory conclusions. In this study, we collected a total of 10,826 cases from 186 publications in the China National Knowledge Infrastructure Database to quantitatively and systematically investigate the general patterns and driving factors of power generation performance in MFCs. Our results showed that (1) the power density, voltage, and reaction duration were significantly lower (~25%) in this study, while the coulombic efficiency and ambient temperature were significantly higher (13.4–33.1%) than those reported in other meta-analyses or review papers; (2) reaction chamber volume and cathode surface area were stronger predictors for the majority of power generation performance indices than other device configuration indices, especially cathode chamber volume, which explained >70% of the variances in power density and coulombic efficiency; (3) ambient temperature, external resistance, and reaction duration had greater effects on power generation performance than other reaction conditions; and (4) substrates with pre-treatment, especially with biological treatment, showed 10–40% higher values for the majority of power generation performance indices compared to pre-treatment with physical and chemical methods, and solid substrates showed better power generation performance than liquid and fluid substrates for the majority of indices. Our results suggest that dual-chamber systems, larger cathode surface areas, neutral pH levels, ambient temperatures of 30–35°C, and biological pre-treatment of substrates may be helpful in improving the power generation performance of MFCs.

## Introduction

Energy is essential for sustaining human life and supporting its global production, with the majority of countries and regions currently heavily dependent on conventional fossil fuels for energy generation (Slate et al., 2019; Vogt and Weckhuysen, 2024). However, fossil energy is a non-renewable resource, and its combustion, along with the production of various byproducts, invariably results in serious environmental problems, including the release of large amounts of greenhouse gases and numerous toxic organic pollutants (Shindell and Smith, 2019). As a result, the development of new clean energy technologies and the restructuring of the energy sector have become global priorities for sustainable development (Green et al., 2024). The capability of microbial fuel cells (MFCs) to use electricity-producing microbes to convert chemical energy in organic matter into electricity has made them one of the most promising new energy technologies in the field of ecology and environmental science (Pandey et al., 2024; Du et al., 2007). This is especially important in modern society, as we face the challenge of treating large quantities of sludge and polluted sediments in aquatic ecosystems (Li et al., 2017). Given that MFCs can utilize industrial wastewater, household wastewater, and many other kinds of organic pollutants as substrates for microbial growth, they represent a promising technology for addressing the problem of excessive sludge in the context of rapid urbanization, especially in developing countries (Krieg et al., 2019).

Research on MFCs has rapidly advanced since the 1990s due to their dual capabilities of power generation and pollutant removal (Zhang et al., 2016, 2022; Xu et al., 2016; Cao et al., 2021). Over >11,000 papers related to laboratory MFC experiments have been published worldwide (Bird et al., 2022). MFCs can effectively remove various pollutants: 60–97% for heavy metals such as Cd, Zn, and Cu (Abourached et al., 2014); >50% for emerging contaminants (e.g., microplastics, antibiotics, and personal care products; Li et al., 2023; Qin et al., 2021); 60–99% for organic pollutants such as COD (Zhang et al., 2015); and 20–95% for nitrogen and phosphorus (Tao et al., 2025; Ge et al., 2020; Gao et al., 2024; Hu et al., 2020). Many researchers have sought to enhance electricity generation performance, not only for its energy benefits but also because it is closely linked to the effectiveness of pollutant removal. However, many factors (e.g., pH, electrode material, and ambient temperature) can affect the power generation performance of MFCs, which limits their widespread application (Chakma et al., 2025; Jalili et al., 2024). Therefore, identifying the key factors that affect the power generation performance of MFCs is crucial.

The power generation performance of MFCs is generally represented by indicators, including voltage, current, current density, power density, and coulombic efficiency, and these indices vary significantly among studies (Sonawane et al., 2017). Empirical studies have indicated that the majority of MFCs typically have voltages between 0.2 and 1.2 V and currents between 1 and 10 mA (An et al., 2016; Prasad and Tripathi, 2022). Although the current density measured in MFC experiments ranges from 0 to > 10 A/m^2^, more than half of the studies reported relatively narrow ranges: 0.085–1.3 A/m^2^ (Fouchecour et al., 2022). The power density of MFCs has increased from 0.001 mW/m^2^ in the early stages to 250–740 mW/m^2^ currently, and even as high as 6,860 mW/m^2^ in specific investigations (Bird et al., 2022; Sun et al., 2022). For volumetric power density, the majority of studies reported values ranging from 0 to 0.5 W/m^3^, with only a small fraction (1.5%) of studies achieving 100–420 W/m^3^.

However, these values are still much lower than those of conventional Li-ion dry batteries (90 kW/m^3^; Fouchecour et al., 2022). Coulombic efficiencies are generally low in the majority of studies, ranging from <1% to 20% (Yu et al., 2023; Sonawane et al., 2022), although some studies reported values as high as 20–60% (Chen et al., 2021; Zafar et al., 2022), indicating that electrons may be consumed in other reactions and that microbes in the MFCs were less effective at converting electrons into current (Bird et al., 2022). These studies suggest that the power generation performance of MFCs is relatively low in magnitude and stability, especially when compared to that of lithium, lead-acid, and other conventional batteries.

Generally, the factors that significantly influence the power generation performance of MFCs can be divided into three categories: reaction conditions, substrate properties, and MFC device configurations (Du et al., 2007; Abubackar et al., 2023; Mahmoudi et al., 2024). First, electrode surface area, reaction chamber volume, and electrode type are important device configurations that have strong effects on the power generation performance of MFCs (Song et al., 2022; Shi et al., 2025). The majority of MFC studies have used either single-chamber or dual-chamber systems, each with its own advantages and disadvantages (Zadeh et al., 2024; Khan et al., 2018). Due to the limited reaction chamber volume and electrode surface area, the output current of many small MFCs usually falls within the range of 1–10 mA (Tee et al., 2018). The reaction chamber volume of the majority of MFCs ranges from 0 to 50 L, and current density may decrease with increasing reaction chamber volume (Bird et al., 2022). Multiple small-sized MFCs are typically employed in series or parallel in actual wastewater treatment plants because a single large-volume MFC may exhibit low power generation performance due to the considerable distance between the anode and cathode chambers (An et al., 2014; Ge and He, 2016). The current, voltage, and coulomb efficiency of MFCs can also be directly affected by the characteristics of electrode materials (Hindatu et al., 2017; Aiswaria et al., 2022). For example, materials with high conductivity, such as graphite and carbon fiber paper, can increase coulombic efficiency by improving electron transport (Logan et al., 2007). The anodic material and geometry are also recognized as influential factors for MFC performance. Graphite is the most widely used anodic material (83% of anodes) due to its advantages, such as high specific surface area, excellent electrical and thermal conductivity, low cost, and mechanical strength (Olabi et al., 2020; Aiswaria et al., 2022). The geometrical characteristics of anodes strongly influence the surface-to-volume ratio, which positively affects the working volume and the electroactive biofilm. Brush and granular electrodes are commonly used as anodic material for small-scale MFCs, offering relatively high surface-to-volume ratios (86 and 106 m^2^/m^3^, respectively; Fouchecour et al., 2022). MFCs with brush anodes exhibited 130% higher power density than those with flat surface anodes (Zafar et al., 2022).

Secondly, substrate characteristics, especially the species and activity of microbes in the substrate, play an important role in the electricity production performance of MFCs, which primarily rely on microbial metabolic processes to convert chemical energy into electrical energy (Sonawane et al., 2022; Hou et al., 2011). Various types of inocula and substrates have been used in MFC experiments to establish the anodic biofilm, with activated sludge being the most commonly used aerobic inoculum (Obata et al., 2020; Wei et al., 2024). The electricigens, which directly affect the power generation performance of MFCs, can be sourced from any sludge, sediment, or wastewater. Commonly identified microorganisms include *Shewanella, Geobacter*, and some fungi species (e.g., *Saccharomyces cerevisiae*), and the total number of electricigen species may exceed several hundred (Zhang et al., 2017; Logan et al., 2019). The operating phases of substrates usually include liquid, fluid, and solid forms, with solid substrates showing >38% higher coulombic efficiency than liquid substrates, although power density (including volumetrically normalized values) did not differ significantly between the two phases (Zafar et al., 2022). Another influential substrate factor affecting power generation performance is the concentration of organic matter. For example, when the concentration of COD in the substrate reaches a certain threshold, it is negatively correlated with coulombic efficiency and positively correlated with current density (Dowdy et al., 2018). Furthermore, a substantial decrease in the persistence of MFC current, voltage, and coulombic efficiency may result from microbial metabolic activities being affected by factors such as depleted or insufficient substrate supply and elevated concentrations of toxic pollutants during prolonged operation (Bird et al., 2022; Di Lorenzo et al., 2014).

Finally, the power generation performance of MFCs is also influenced by reaction conditions, including ambient temperature, dissolved oxygen, hydraulic retention time, and pH (Sorgato et al., 2023; Wei et al., 2013). Environmental conditions significantly affect microorganisms (in terms of species abundance and richness) and chemical reaction processes, thereby influencing MFC performance (Jia et al., 2014). However, reaction conditions such as temperature and pH have not been examined in many MFC studies (Bird et al., 2022). The pH condition in the majority of studies was around 7, which falls within the optimal operational range for the majority of anodic microbes (Wang et al., 2016; Margaria et al., 2017). MFCs operating at neutral pH showed a higher median coulombic efficiency (19%) compared to those in acidic (14%) and alkaline (11%) conditions (Fouchecour et al., 2022). However, proton accumulation in the anode can lead to acidification, which may inhibit oxidation and fermentation activities (Babanova et al., 2020). High pH levels in the cathodic chamber, resulting from the depletion of protons and the accumulation of hydroxyl anions, also significantly affect MFC performance (Lu et al., 2017). Regarding temperature, although MFCs can operate at outdoor temperatures ranging from −10 to 35°C, some studies have identified ideal ambient temperatures of 30–40°C or even up to 95°C (Lin et al., 2024; Fu et al., 2015). The majority of studies, however, were conducted at temperatures between 20°C and 35°C, which falls within the active temperature range for the majority of electrogenic bacteria (Song et al., 2017; Wei et al., 2013). The performance of MFC usually increases with rising outdoor temperatures (Liu et al., 2005). Nevertheless, reported outdoor ambient temperatures may be misleading, as they do not reflect the actual operating temperatures, and discrepancies may exist between startup and operational temperatures (Liu et al., 2012). Other factors, such as variations in hydraulic retention time, also influence power generation performance through their effects on microbiome performance. A moderate retention duration (e.g., 1–2 days) is recommended for achieving optimal power performance (Castellano-Hinojosa et al., 2024; Haavisto et al., 2017).

In this study, we focused on the characteristics of the key parameters of MFC power generation performance and its driving factors. We collected data from 186 publications in the China National Knowledge Infrastructure (CNKI) Database to systematically investigate (1) the general characteristics of MFCs and comparisons with the results from international counterparts; (2) the fundamental state of MFC device configurations and associated power generation performance indices in China; and (3) the primary factors influencing MFC power generation performance.

## Materials and methods

### Dataset and data extraction

**Figure 1:**
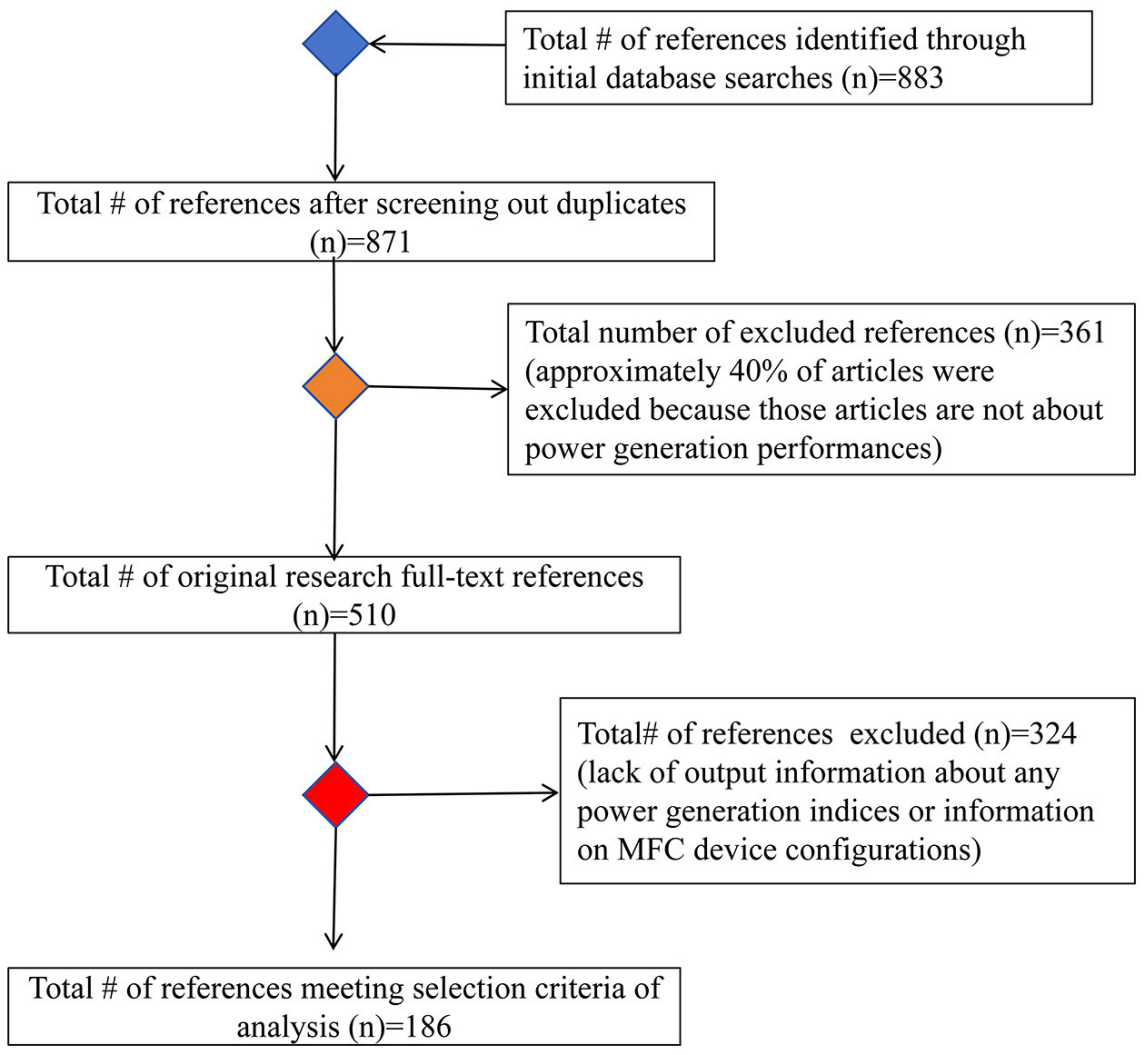
The data collection and screening scheme in this study.

To gain a clear understanding of the current state and to identify the driving factors influencing the power generation performance of MFCs in China, a comprehensive database was built by searching the CNKI database using the following terms: “(microbial fuel cell) AND (power generation performance OR coulombic efficiency OR current OR voltage OR power density OR current density)” in the keyword, abstract, and title fields to retrieve relevant literature (data were updated prior to 30th November, 2024). However, the inclusion of only Chinese publications may not fully represent all MFC studies conducted in China. Nevertheless, our dataset remains meaningful, as many researchers publish papers in both Chinese and English, and some papers may share the same data derived from a common series of experiments, especially regarding device configurations, reaction conditions, and substrate characteristics. To ensure the quality of our dataset, we excluded publications that had not been peer-reviewed (e.g., academic dissertations). Initially, we selected 883 publications, which were carefully screened based on specific criteria to ensure their suitability and relevance. First, the selected MFC studies had to be experimental, excluding review papers. Secondly, they had to report at least one index of power generation performance (e.g., voltage, power density, or coulombic efficiency). Finally, these studies had to provide at least one index related to MFC device configurations, reaction conditions, or substrate characteristics. After rigorous screening and evaluation (Figure 1), our final dataset comprised 10,826 cases drawn from 186 publications (Appendix I). For each publication, we collected data on power generation performance (current, voltage, power density, current density, and coulombic efficiency) and three categories of potential influential factors: device configurations (e.g., anode surface area, chamber volume, and cathode surface area); reaction conditions (e.g., ambient temperature, pH, and external resistance); and substrate characteristics (e.g., pre-treatment type and operating phase). Data on MFC power generation performance and the potential driving factors were either directly collected from tables and manuscripts or indirectly extracted from figures using an online tool (https://plotdigitizer.com/app). We grouped the operating phases of substrates into three categories: liquid, solid, and fluid. We also classified the pre-treatment types into biological, chemical, physical, and multiple methods (e.g., biochemical and physical chemical). In addition, to enable comparisons with international counterparts, we also collected data from relevant meta-analyses or review papers (Appendix II).

### Data analysis

First, to compare the results between our study and other meta-analyses or review papers, *t*-tests (for voltage and reaction duration) and one-way ANOVA (for power density, temperature, and coulombic efficiency) were conducted based on the number of studies with available data. For one-way ANOVA, *post hoc* multiple comparisons were performed using Tukey's test (assuming equal variance) and Dunnett's T3 test (assuming unequal variance) when differences were significant (*P* < 0.05). Then, the sample size, mean, median, extreme value, coefficient of variation (CV), and other indices were calculated for all collected data to examine general patterns. All potential influential factors were grouped into three categories: device configurations, reaction conditions, and substrate characteristics. In order to test the effects and strengths of each potential influential factor on each power generation performance index, *t*-tests (for reaction chamber type and substrate pre-treatment) and one-way ANOVA (for substrate type) were conducted for categorical variables, and correlation analyses were conducted for continuous variables. Pearson and Spearman correlations were used for parametric and non-parametric data (the latter only for current), respectively. We also tested non-linear relationships and compared them with linear relationships. Non-linear models were selected when *R*^2^ values were substantially higher than those of linear models. For each group of potential, influential categorical factors, if a focal power generation performance index was significantly correlated with two or more continuous factors, a multiple linear regression model was conducted to identify the main contributors. Finally, a generalized linear model was used to investigate whether there were interactions between categorical variables and continuous variables identified by the multiple linear models. Before conducting these analyses, the normality, outliers, and homogeneity of variance of all data were examined, and data were transformed to meet the assumptions of normality and homogeneity (see Supplementary Tables S1, S2 for details of data transformation). Indices collected from fewer than three studies or with fewer than 30 cases were excluded from all tests. All statistical analyses were conducted using IBM SPSS Statistics 26.0, and all figures were generated using Origin 2025b.

## Results

### Comparisons of this study with other meta-analyses and review papers

**Figure 2:**
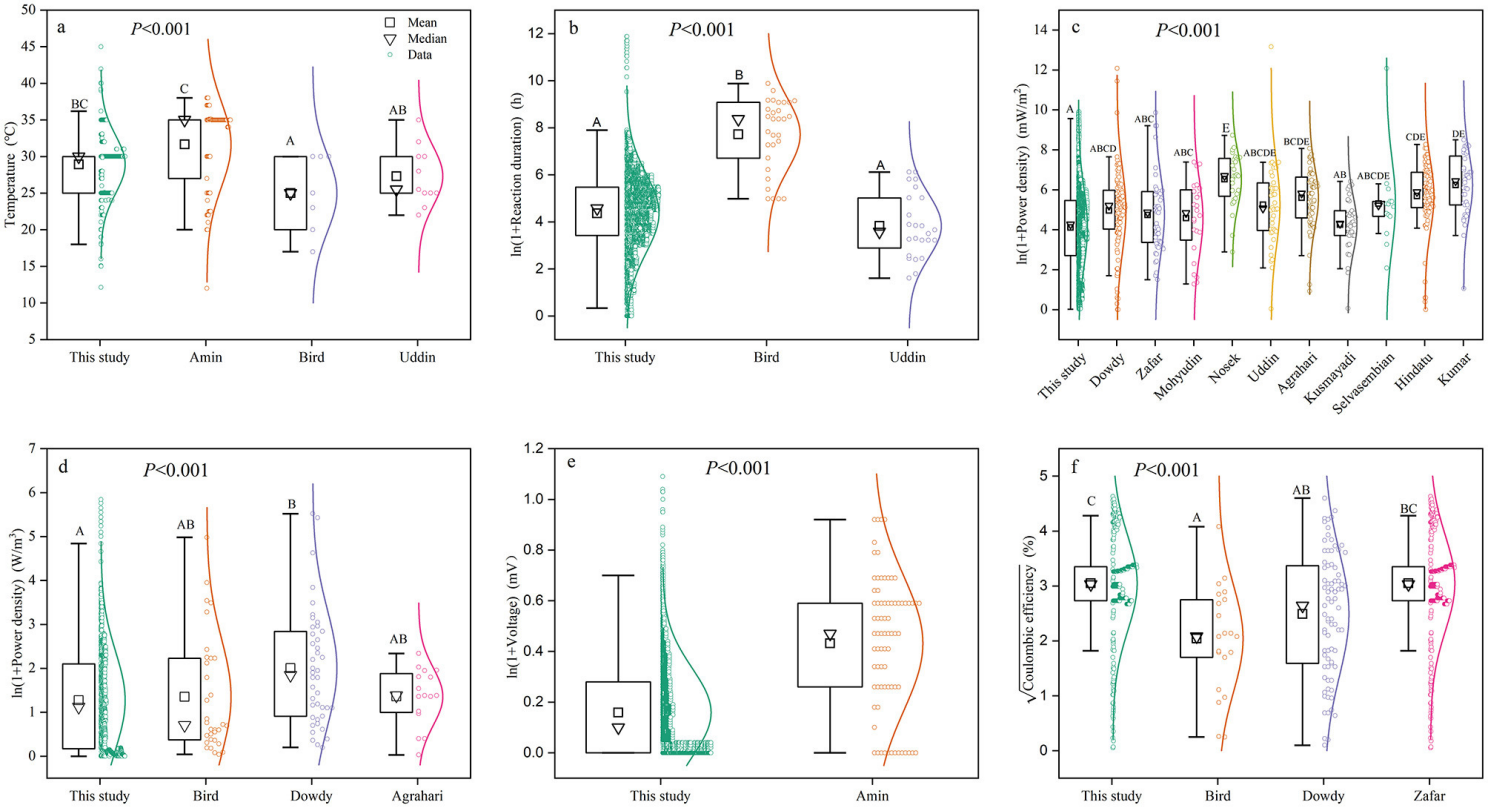
Comparisons of power generation performance among different studies (please see reference details in Appendix II): **(a)** temperatures; **(b)** reaction duration; **(c)** power density (mW/m^2^); **(d)** volumetric power density (W/m^3^); **(e)** voltage; and **(f)** coulombic efficiency. Different capital letters following the data in the table indicate significant differences (*p* < 0.05). That is, the differences between treatment groups with the same letter are not significant, and the differences between treatment groups with different letters are significant.

We found generally lower power density, voltage, and reaction duration but higher operating temperature and coulombic efficiency in our study compared to other studies (Figure 2). More specifically, the power density in our study (59.95 ± 6.11 mW/m^2^) was significantly lower than that in other studies (Figure 2c). The volumetric power density in our study (2.56 ± 2.03 W/m^3^) was only 60.2 % of that reported by Dowdy et al. (2018). However, it showed no significant difference compared to the values reported by Bird et al. (2022) and Agrahari et al. (2022). Coulombic efficiency significantly differed among studies (Figure 2f); our study reported a value of 9.30 ± 0.76%, which was 18.4 and 33.1% higher than those reported by Dowdy et al. (2018) and Bird et al. (2022), respectively, but showed no significant difference from Zafar et al. (2022). Moreover, significant differences in voltage were observed among studies (Figure 2e), with our study recording 182.69 ± 2.16 mV, which was 19.8% lower than the values reported by Amin et al. (2022). Regarding reaction conditions, the reaction duration in our study (78.04 ± 4.31 h) was lower than that reported in the study by Bird et al. (2022) but showed no significant difference compared to Uddin et al. (2021). Our study also reported relatively higher temperatures than those in the other studies (Figure 2a).

### General patterns of MFC characteristics and power generation performance

The general information on the mean, median, maximum, minimum, and coefficient of variation (CV) for all data is shown in Table 1. The majority of CV values fell within the range of 20–40%, with the exception of power, which had a CV as high as 306.61%, and ambient temperature, which had a CV of only 4.32%. Power density and current density also exhibited relatively high CV values (87–89%), while external resistance showed a relatively low CV (11.55%). CV values were generally high for power generation performance indices (>40%), while they were typically low for device configurations (<40%). The majority of data showed relatively small variances between mean values and median values, except for internal resistance, for which the mean was 15.1% higher than the median value. However, the relatively low CV values may be due to the data transformations applied.

**Table 1:** Basic information on MFC device configurations and power generation performance in this study.

**MFC**	**Parameters**	**Mean ±SD**	**Max**	**Min**	**Med**	**CV**
Power generation performance	Voltage (mV)	5.219 ± 1.129	7.93	1.10	5.46	21.63 %
	Power (W)	−0.772 ± 2.367	3.40	−8.57	−0.20	−306.61 %
	Power density (mW/m^2^)	4.111 ± 1.953	9.91	0.02	4.27	47.51 %
	Power density (W/m^3^)	1.279 ± 1.129	6.28	0.00	1.13	88.27 %
	Coulombic efficiency (%)	4.796 ± 1.925	9.34	0.23	4.43	40.14 %
	Current density (mA/m^2^)	4.526 ± 2.164	8.72	0.00	4.90	47.81 %
	Current density (A/m^3^)	2.300 ± 2.009	8.40	0.00	2.32	87.35 %
MFC device configurations	Anode material area (cm^2^)	3.354 ± 1.119	8.19	0.02	3.26	33.36 %
	Cathode material area (cm^2^)	3.396 ± 1.374	8.19	0.02	3.26	40.46 %
	Cathode chamber volume (mL)	5.611 ± 1.574	9.21	0.05	5.48	28.05 %
	Anode volume (mL)	5.491 ± 1.473	12.41	0.92	5.17	26.83 %
	Total volume of reaction chamber (mL)	6.035 ± 1.990	13.22	0.10	5.99	32.97 %
Reaction conditions	Battery start-up time (d)	2.712 ± 0.927	5.39	0.00	2.61	38.18 %
	Reaction duration (h)	4.357 ± 1.647	11.59	0.00	4.56	37.80 %
	pH	48.907 ± 11.852	81.00	14.44	49.00	24.23 %
	Cathode pH	1.641 ± 0.426	2.48	0.00	1.95	25.96 %
	Temperature (°C)	3.356 ± 0.145	3.91	2.71	3.40	4.32 %
	Internal resistance (Ω)	7.138 ± 3.027	14.57	0.00	6.20	42.41 %
	External resistance (Ω)	6.772 ± 0.782	9.21	4.28	6.91	11.55 %

All data in the table were transformed. For the specific conversion, refer to Supplementary Table S1.

**Figure 3:**
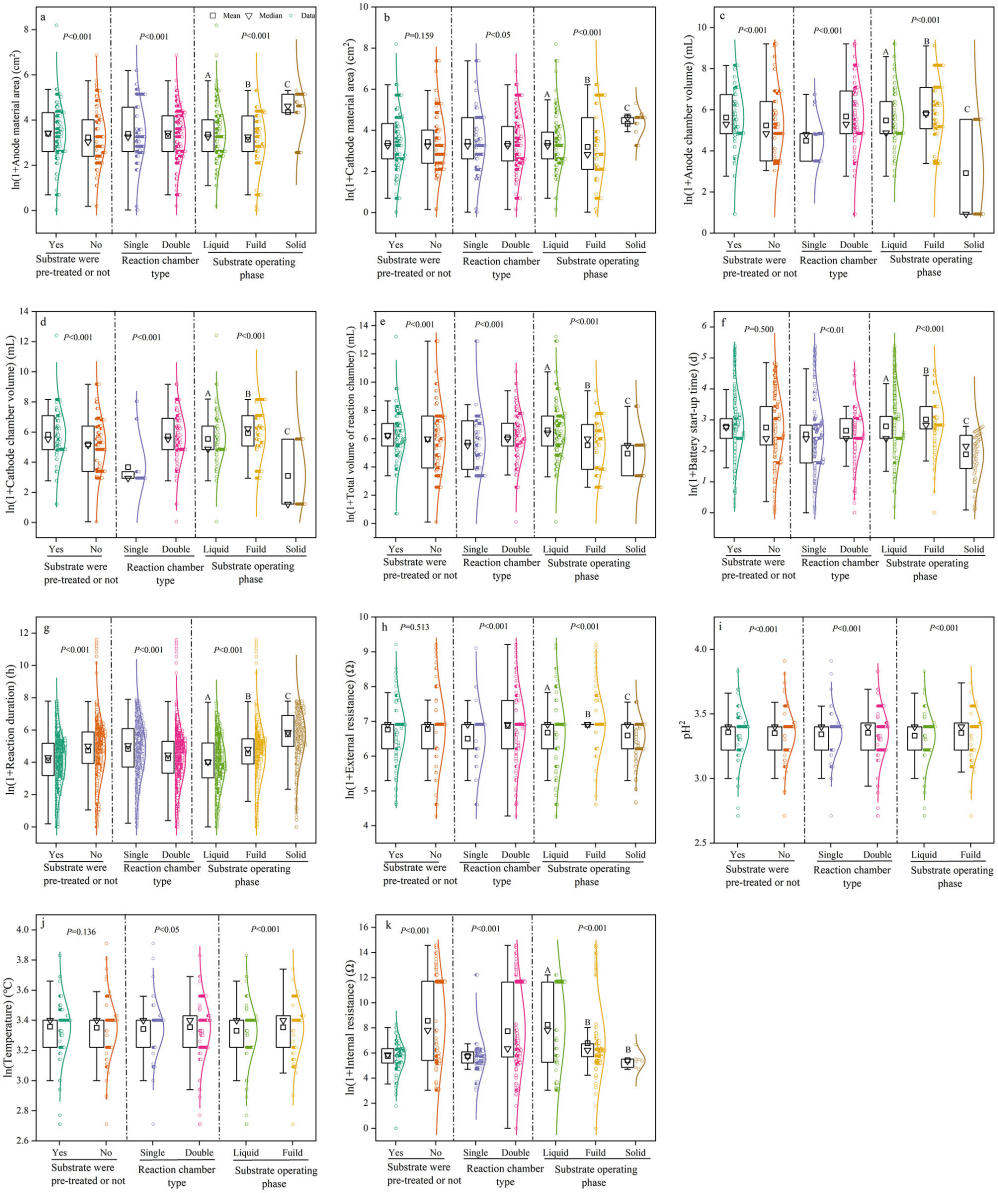
Box plots comparing the effects of substrate pre-treatment, chamber type, and substrate type (operating phase) on MFC device characteristics and reaction conditions. MFC device characteristics included **(A)** Anode material area; **(B)** Cathode material area; **(C)** Anode chamber volume; **(D)** Cathode chamber volume; and **(E)** Total volume of reaction chamber. Reaction conditions included **(F)** Battery start-up time; **(G)** Reaction duration; **(H)** External resistance; **(I)** pH; **(J)** Temperature; and **(K)** Internal resistance. Different capital letters following the data in the table indicate significant differences (*p* < 0.05). That is, the differences between treatment groups with the same letter are not significant, and the differences between treatment groups with different letters are significant.

The characteristics of MFC devices were influenced by reaction chamber type, substrate pre-treatment, and substrate operating phase (Figure 3). Dual-chamber MFCs exhibited 4.7–36.8% higher values for the majority of device characteristics compared to single-chamber MFCs. However, dual-chamber MFCs had 3.0 and 12.1% lower electrode surface area and reaction duration, respectively, than single-chamber MFCs. Reaction duration, pH, and internal resistance were 12.9%, 3.7%, and 32.7% lower, respectively, in MFCs with substrate pre-treatment compared to those without pre-treatment. In contrast, other device characteristics were higher in MFCs with pre-treatment than in those without. We also found that fluid and solid substrates had strong effects on the majority of MFC device characteristics, with the exception of internal resistance. For start-up time, reaction chamber volume, external resistance, and pH, MFCs with fluid substrates showed values that were 6.6–11.9% and 37.4–50.1% higher than those with liquid and solid substrates, respectively. Regarding electrode surface area, reaction duration, and temperature, MFCs with solid substrates had 23–25% higher values than those with fluid and liquid substrates.

### Factors affecting the power generation performance of MFCs

**Figure 4:**
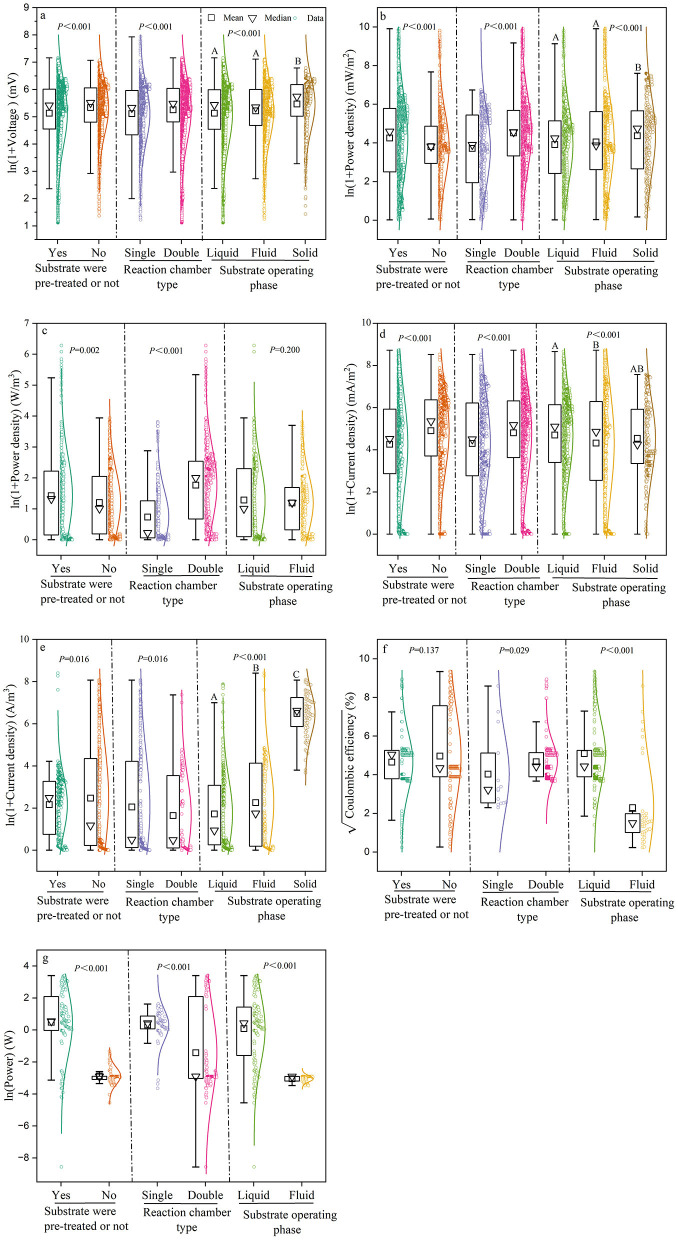
Box plots comparing the effects of substrate pre-treatment, chamber type, and substrate type (operating phase) on the power generation performance of MFCs. power generation performance of MFCs included **(a)** Voltage; **(b)** Power density (mW/m^2^); **(c)** volumetric power density (W/m^3^); **(d)** current density (mA/m^2^); **(e)** volumetric current density (A/m^2^); **(f)** Coulombic efficiency; and **(g)** Power. Different capital letters following the data in the table indicate significant differences (*p* < 0.05). That is, the differences between treatment groups with the same letter are not significant, and the differences between treatment groups with different letters are significant.

The results of the *t*-test and one-way ANOVA showed that the power generation performance of MFCs was significantly affected by reaction chamber type, substrate operating phase, and pre-treatment (Figure 4). The power density of MFCs without substrate pre-treatment was 44.02 ± 4.54 mW/m^2^ and 2.33 ± 1.79 W/m^3^, which were 10.5 and 15.5% higher, respectively, than those of MFCs with pre-treatment. By contrast, the current density of MFCs without substrate pre-treatment was 133.96 ± 5.94 mA/m^2^ and 10.79 ± 11.47 A/m^3^, which were 13.1 and 12.3% lower, respectively, than in MFCs with pre-treatment. Moreover, voltage followed the same trend, decreasing by 4.9% after pre-treatment. Dual-chamber MFCs showed ~15% higher values in the majority of electricity generation indices compared to single-chamber MFCs, except for voltage and power. The voltage in dual-chamber MFCs was only 3% higher than that in single-chamber MFCs, whereas the power output was 80% higher. The power density and voltage of MFCs using solid substrates were 77.73 ± 6.28 mW/m^2^ and 235.99 ± 1.53 mV, respectively, which were 10 and 7% higher than those using liquid and fluid substrates. However, volumetric power density showed no significant differences among substrate operating phases. The coulombic efficiency of MFCs with liquid substrates was 25.92 ± 2.79%, which was nearly half that of solid substrates. Current density was highest (108.73 mA/m^2^) for liquid substrates, while volumetric current density was highest (643.19 A/m^3^) for solid substrates.

**Figure 5:**
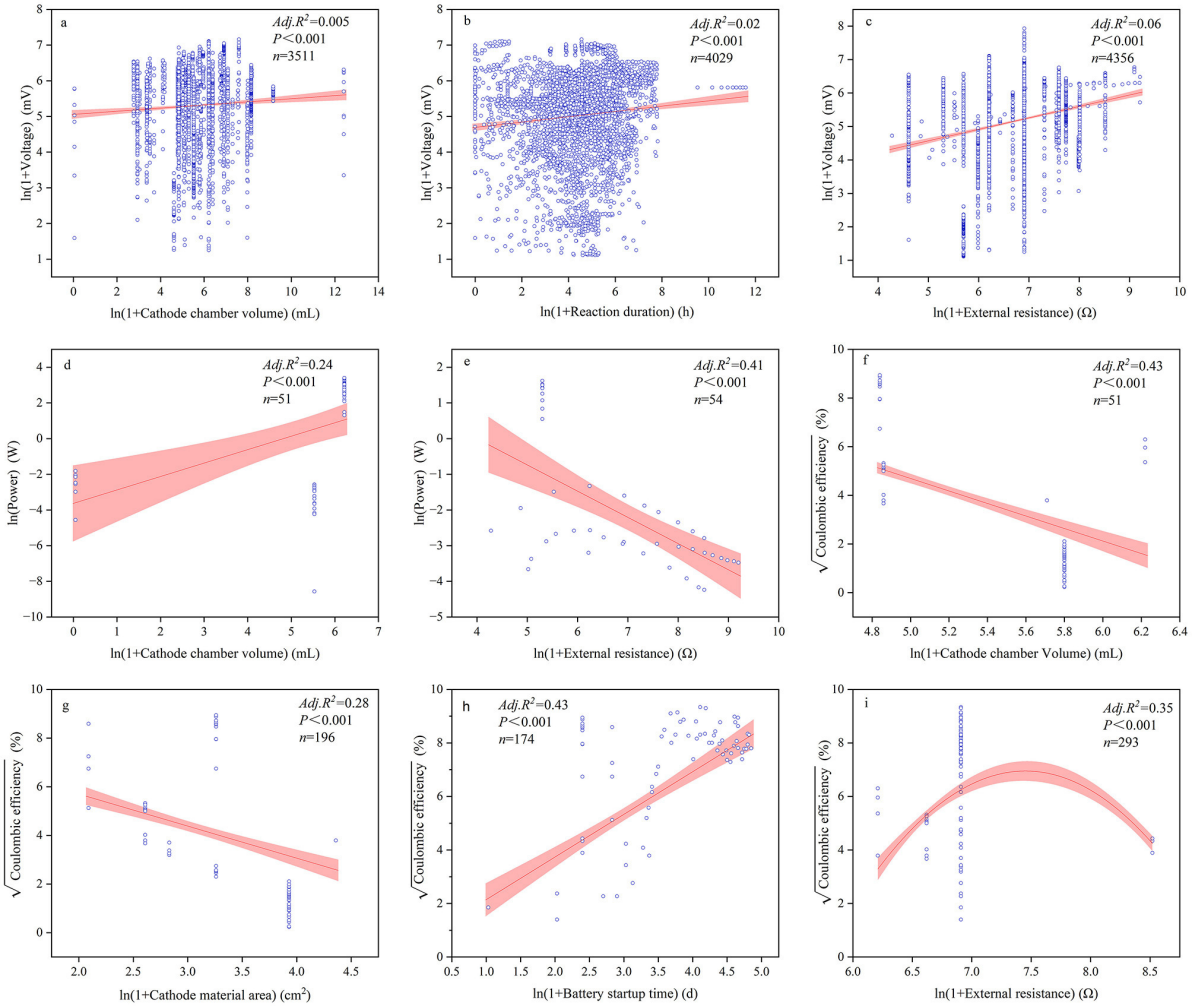
Relationships between voltage **(a–c)**, power **(d–f)**, and coulombic efficiency **(g–i)** alongside significant influencing factors.

**Figure 6:**
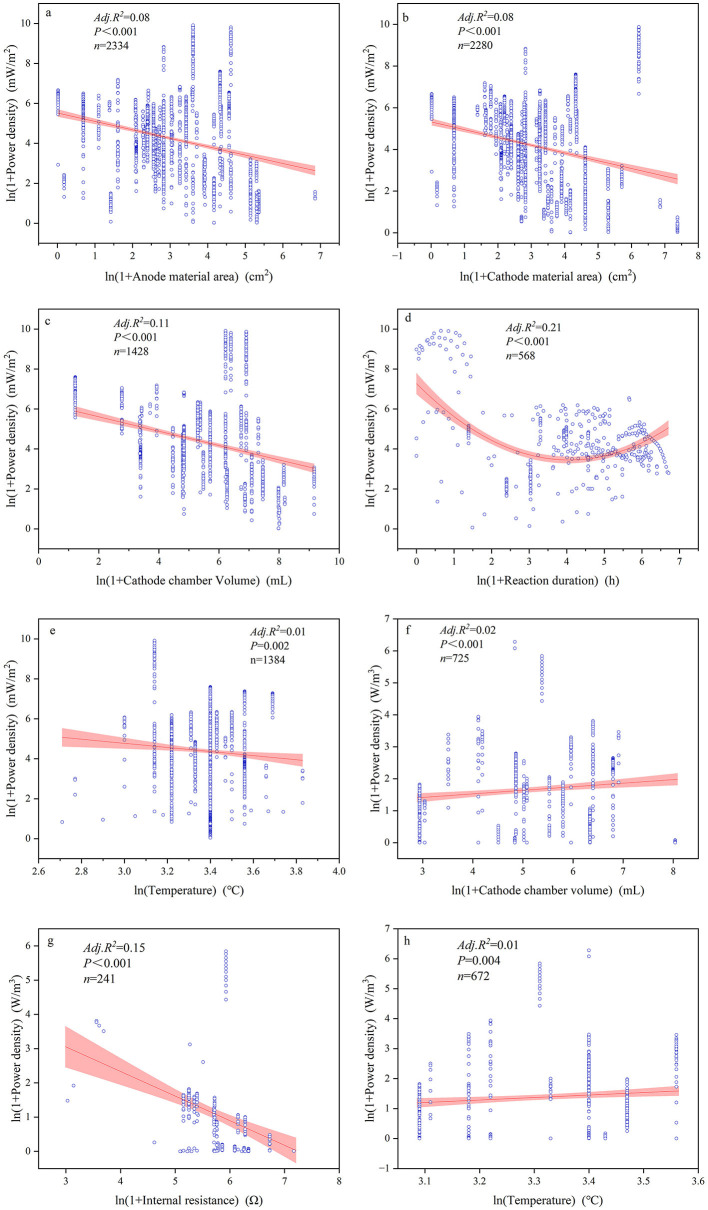
Relationships between power density **(a–e)** and volumetric power density **(f–h)** with significant influential factors.

**Figure 7:**
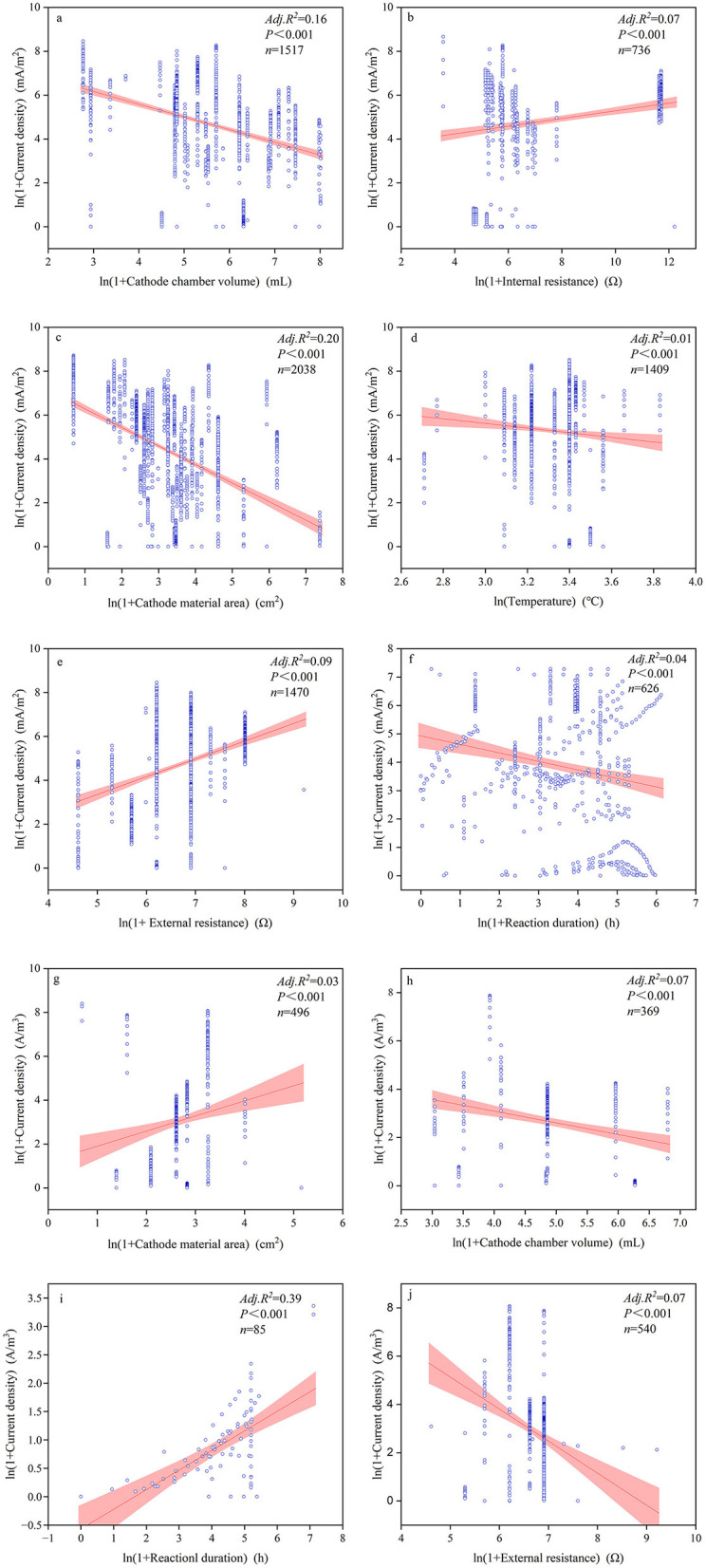
Relationships between current density **(a–f)** and volumetric current density **(g–j)** with significant influential factors.

The majority of continuous variables related to device configurations and reaction conditions had significant impacts on power generation performance (Figures 5–7, Supplementary Table S3). Among these, the most influential factors were cathode chamber volume and cathode surface area. Cathode surface area significantly affected all power generation performance indices, except voltage, with R^2^ values ranging from 0.01 to 0.257. It had the lowest explanatory power for power output but the highest for coulombic efficiency (*R*^2^ = 0.257). Furthermore, the cathode surface area exhibited the highest positive correlation with power (*R* = 0.313) and the highest negative correlation with coulombic efficiency (*R* = −0.529). Another important device configuration factor was cathode chamber volume, which showed the lowest explanatory power for voltage (*R*^2^ = 0.01) but explained 43.1% of the variance in coulombic efficiency. Similar to the cathode surface area, cathode chamber volume showed the strongest positive correlation with power (*R* = 0.493) and the strongest negative correlation with coulombic efficiency (*R* = −0.653). Other noteworthy factors included pH and reaction duration. Reaction duration significantly influenced all power generation performance indices except for coulombic efficiency, with *R*^2^ values ranging from 0.01 to 0.59. It had the weakest explanatory power for voltage (1.0%) and the strongest for power (59.5%). Specifically, our result showed that power density was negatively correlated with reaction duration between 0 and 32.11 h and positively correlated between 32.11 and 1,095.63 h (*R*^2^ = 0.21). Moreover, pH significantly affected all indices except volumetric current density (A/m^3^), with *R*^2^ values ranging from 0.01 to 0.59. It was negatively correlated with power density (*R*^2^ = 0.116) but showed positive effects on the other power generation performance indices, explaining 85.1% of the variance in coulombic efficiency.

The multiple regression analysis showed that among all device configurations, the most important factors were cathode surface area and cathode chamber volume (Table 2). Cathode chamber volume significantly affected all power generation performance indices, with the highest explanatory power for volumetric power density (R^2^ = 0.813) and the lowest for current density (*R*^2^ = 0.128). Regarding the cathode material area, it affected power density, current density, and coulombic efficiency, with *R*^2^ values ranging from 1.0 to 33.4%. Among all electricity generation performance indices, device configurations showed the best goodness of fit for coulombic efficiency (*R*^2^ = 0.891). For reaction conditions, the most important factors were reaction duration and external resistance. Reaction duration had the weakest explanatory power for voltage (*R*^2^ = 0.014) but the strongest for volumetric current density (*R*^2^ = 0.759). External resistance was significantly correlated with all electricity generation performance indices except power density, with a maximum *R*^2^ value of 0.742. Among all electricity generation performance indices, reaction conditions showed the best goodness of fit for volumetric current density (*R*^2^ = 0.785). When considering both device configurations and reaction conditions together, the best-fitting model was for coulombic efficiency (*R*^2^ = 0.941). Notably, both device configurations and reaction conditions explained <5% of the variance in voltage. In addition, continuous and categorical variables showed complex interaction effects on electricity generation performance (Supplementary Table S4). For the majority of electricity generation performance indices, if the main effects were significant, the interaction effects were also significant. However, even if the main effects were not significant, the interaction effects may still be significant. For example, the effect of the substrate operating phase on current density was not significant on its own; however, when the cathode surface area was considered, the interaction effect became significant.

**Table 2:** Results of multiple linear regressions for each power generation performance indicator and important influential factors.

**Power generation performance**	**Factors**	**Variables**	**Regressions**	***F***	**R^2^**	**Excluded variables**
Power density (mW/m^2^)	Characteristics of device	AMA (13.2), CMA (15.7), CCV (15.8)	ln (PD+1) = 6.341 + 1.644 ln (1+CMA)−0.526 ln (1+CCV)−1.483 ln (1+AMA)	*F*_3, 1106_ = 298.220^***^	0.447	ACV, BIR
	Reaction conditions	RD (34.9), Tem (7.3)	ln (PD+1) = 15.632–0.344 ln (1+RD)−2.838 ln (1+Tem)	F_2, 356_ = 131.185^***^	0.765	pH
	All	RD (37.1), CCV (7.1), Tem (0.8)	ln (PD+1) = -7.682–3.555 ln (1+RD) + 0.918 ln (1+CCV)+2.859 ln (1+Tem)	F_3, 301_ = 82.762^***^	0.672	AMA, CMA
Power density (W/m^3^)	Characteristics of device	BIR (6.2), CCV (81.3)	ln (PD+1) = −0.201 + 1.788 ln (1+CCV)−0.705 ln (1+BIR)	F_2, 166_ = 580.666^***^	0.875	AMA, CMA, ACV, BIR
	Reaction conditions	Tem (56.5)	ln (PD+1) = 11.090–3.244 ln (1+Tem)	F_1, 239_ = 310.270^***^	0.565	pH, RD, ER
	All	CCV (81.0), BIR (6.4)	ln (PD+1) = −0.139 + 1.796 ln (1+CCV)−0.720 ln (1+BIR)	F_2, 168_ = 584.503^***^	0.874	Tem
Voltage (mV)	Characteristics of device	CCV (0.5)	ln (V+1) = 4.968 + 0.048 ln (1+CCV)	F_1, 1869_ = 9.293^**^	0.005	ACV, AMA
	Reaction conditions	RD (1.9), ER (1.4)	ln (V+1) = 3.489–0.057 ln (1+RD) + 0.280 ln (1+ER)	F_2, 447_ = 7.438^***^	0.032	-
	All	CCV(3.1), RD (1.8)	ln (V+1) = 5.107 + 0.095 ln (1+CCV)−0.074 ln (1+RD)	F_2, 1411_ = 35.884^***^	0.048	ER
Power (W)	Characteristics of device	CCV (24.3)	ln (P+1) = −3.630 + 0.753 ln (1+CCV)	F_1, 49_ = 15.742^***^	0.243	CMA, ACV
	Reaction conditions	ER (41.4)	ln (P+1) = 2.930–0.743 ln (1+ER)	F_1, 52_ = 36.737^***^	0.414	RD
	All	ER (41.3)	ln (P+1) = −1.260–0.302 ln (1+ER)	F_1, 11_ = 7.747^*^	0.413	CCV
Current density (mA/m^2^)	Characteristics of device	CCV (12.8), BIR (11.5), CMA (1.0)	Ln (CD+1) = 4.755–0.420 ln (1+CCV) +0.229 ln (1+BIR) +0.245 ln (1+CMA)	F_3, 576_ = 64.919^***^	0.253	ACV, AMA
	Reaction conditions	Tem (47.5), ER (14.1), RD (7.6)	Ln (CD+1) = −27.228 + 9.260 ln (1+Tem) +0.386 ln (1+ER)+0.182 ln (1+RD)	F_3, 199_ = 148.577^***^	0.691	BST, pH
	All	ER (61.0), RD (4.9)	Ln (CD+1) = −1.630 + 0.924 ln (1+ER)+0.144 ln (1+RD)	F_2, 190_ = 183.593^***^	0.659	CCV, BIR, CMA, Tem
Current density (A/m^3^)	Characteristics of device	CCV (12.9), CMA (15.7)	Ln (CD+1) = 6.850–2.205 ln (1+CCV) +2.629 ln (1+CMA)	F_2, 282_ = 56.560^***^	0.286	AMA, ACV, BIR
	Reaction conditions	RT (75.9), ER (2.7)	Ln (CD+1) = -7.236+ 0.398 ln (1+RD)+0.952 ln (1+ER)	F_2, 47_ = 85.867^***^	0.785	-
	All	CCV (70.3), CMA (3.3), ER (10.5)	Ln (CD+1) = 98.778 + 1.883 ln (1+CCV)−9.42 2ln (1+CMA)−12.128 ln (1+ER)	F_3, 168_ = 297.069^***^	0.841	RD
Coulombic efficiency (%)	Characteristics of device	CCV (55.0), CMA (34.1)	$\sqrt{CE}\;=\;$ 42.857–10.214 ln (1+CCV) +4.450 ln (1+CMA)	F_2, 179_ = 732.722^***^	0.891	AMA, ACV, BIR
	Reaction conditions	BST (63.6), ER (9.6)	$\sqrt{CE}$ = −9.752 + 2.561 ln (1+BST)+0.934ln (1+ER)	F_2, 161_ = 219.527^***^	0.732	pH
	All	BST (93.3), CMA (0.8)	$\sqrt{CE}$ = 19.823–4.277 ln (1+BST)−0.375 ln (1+CMA)	F_2, 36_ = 287.020^***^	0.941	CCV, ER

PD means power density, V means voltage, P means power, CD means current density, CE means coulombic efficiency, AMA means anode material area, CMA means cathode material area, CCV means cathode chamber volume, ER means external resistance, RD means reaction duration, BIR means battery internal resistance, Tem means temperature, BST means battery startup time. ^*^Means *p* < 0.05, ^**^means *p* < 0.01, ^***^means *p* < 0.001.

## Discussion

### Differences in power generation performance between this study and other studies

All power generation performance indices examined in our study differed from those reported in other meta-analyses or review papers. The relatively lower power density in our study may be due to our inclusion of data from the entire stage of the polarization curve for each experiment, while other studies collected only the highest values. The relatively higher coulombic efficiency observed in our study compared to other meta-analyses or review papers may be due to the imbalanced sample size between dual-chamber and single-chamber MFCs. Our dataset included 6,457 cases of dual-chamber MFCs, which was ~2.1 times the number of single-chamber cases. Since the coulombic efficiency of dual-chamber MFCs can be 73% higher than those of single-chamber systems, as supported by our results and other studies (Khan et al., 2018), this imbalance may have contributed to the elevated efficiency. Additional factors contributing to differences between our study and others include variations in sampling size, substrate characteristics (e.g., some studies focused exclusively on food waste; Zafar et al., 2022; Dowdy et al., 2018), and device configurations (e.g., some studies concentrated on anode modification data; Agrahari et al., 2022). Although our study was limited to Chinese papers, which may have caused some bias, our study had clear advantages. Specifically, we compiled a dataset of 11,806 cases, considerably larger than those in other studies (which ranged from dozens to several thousand). In addition, our dataset included a relatively large number of variables, providing a more comprehensive understanding of MFC characteristics and enabling the identification of key driving factors for power generation performance. However, both our study and other review papers reported much lower values for power generation performance metrics—namely voltage, power, power density, current density, and coulombic efficiency—compared to commonly used batteries (e.g., lithium and lead–zinc batteries). This highlights the ongoing challenge of improving the generally low power generation performance of MFCs, which remains a major challenge for future studies (Gupta et al., 2023; Logan and Rabaey, 2012).

### Cathode chamber volume and cathode surface area as key device configuration factors influencing power generation performance

We found that all MFC device configuration variables significantly correlated with at least one power generation performance indicator, with the strength of these relationships ranging from 0.5 to 89.1%. Our results suggest that the effects of device configurations on power generation performance are context-dependent. Among these variables, cathode chamber volume, cathode surface area, and anode surface area emerged as the strongest predictors, as indicated by our linear and multiple regression models. More than 80% of the variances in coulombic efficiency and volumetric power density could be explained by cathode chamber volume and cathode surface area. This result aligns with previous studies, which have also reported positive relationships between power density and both cathode chamber volume and cathode surface area (Opoku et al., 2022; Logan et al., 2007). For example, Cheng and Logan (2011) found that cathode surface area was the most critical factor influencing power density: doubling the cathode surface area increased power by 62% while doubling the anode surface area led to only a 12% increase. A larger cathode surface area provides more room for microbial colonization, thereby enhancing power generation performance (Wang et al., 2024). Additionally, a larger cathode area can increase the rate of the oxygen reduction reaction, a crucial process in MFCs. With greater oxygen availability at the cathode, the overall efficiency of electron transfer improves, leading to a significant increase in power output (Elmekawy et al., 2017). We also observed significant differences in power generation performance between single- and dual-chamber MFCs. Dual-chamber MFCs showed higher values for voltage, power density, current density, and coulombic efficiency, while single-chamber MFCs had higher power output. Our results are in line with those of many other studies (Zafar et al., 2022; Lee et al., 2016). Although dual-chamber MFCs were more frequently studied than single-chamber MFCs, this does not necessarily imply superior performance across all indices. Other influencing factors—such as organic matter loading rate, substrate pre-treatment, and substrate type—may cause extremely high or low power density (Zafar et al., 2022). Notably, we found that cathode chamber volume may exert a stronger effect on power generation performance than anode chamber volume. This implies that the common practice of designing MFCs with equal anode and cathode chamber volumes may need reconsideration to optimize power output (Qian et al., 2009).

Electrical production performance was also affected by chamber type. MFCs with dual chambers exhibited higher voltage, power density, current density, and coulombic efficiency than single-chamber MFCs, partially confirming previous studies (Zafar et al., 2022; Jia et al., 2008). The relatively high coulombic efficiency in dual-chamber MFCs compared to single-chamber MFCs is likely due to the higher levels of dissolved oxygen in single-chamber systems, which may adversely affect microbial activities (Nimje et al., 2012), as well as shifts in microbial communities resulting from prolonged oxygen diffusion in single-chamber MFCs (Flimban et al., 2019). Although we found lower internal resistance in single-chamber MFCs, which should have led to higher power density, we still observed lower power density compared to dual-chamber systems, suggesting that other contributing factors are involved. For example, Lee et al. (2016) found that the abundance of Proteobacteria was 2–3 times higher in dual-chamber MFCs than in single-chamber MFCs and that the dominant archaeal species differed between the two types. Regarding power, the relatively small dataset (177 cases) may have introduced bias, which should be taken into consideration.

### Influences of reaction conditions on the power generation performance of MFCs

Our results indicated that ambient temperature, external resistance, reaction duration, and pH showed stronger effects on the power generation performance of MFCs than other reaction conditions. These results are consistent with previous studies (Pasupuleti et al., 2015; Zhao et al., 2014; Zhang et al., 2010). Ambient temperature significantly affected the power production performance of MFCs. We found that the optimal ambient temperature for maximum power generation performance was 28°C, which falls in the lower range (25–35°C) reported in other studies (Uddin et al., 2021). The majority of studies have shown positive effects of temperature on MFC performance, while performance declines when the temperature exceeds a certain threshold (Hiegemann et al., 2016; Li et al., 2013). This is because microbial activity generally increases with temperature, and many chemical reactions involved in MFC operation also perform optimally at 30–35°C (Zhao et al., 2014). Changes in temperature not only significantly affect the oxidation rates of organic matters but also influence the species diversity (richness and evenness) of anode biofilms, which, in turn, affects metabolic activity, biofilm accumulation, and extracellular electron transfer—all of which impact MFC power generation (Mei et al., 2017). In addition, changes in temperature can affect internal resistance and pH (Pasupuleti et al., 2015). For example, Li et al. (2013) found that internal resistance was approximately 29 Ω at 37°C, but it increased by 62%, 303%, and 48% at 30°C, 10°C, and 43°C, respectively. Other reaction conditions—such as external resistance, internal resistance, and reaction duration—also affected the power generation performance of MFCs. According to Grondin et al. (2012), MFCs are typically operated with a constant exterior resistance, but their internal resistance varies over time. This mismatch reduces power generation efficiency. The highest power output is generally achieved when external resistance matches the total internal resistance (Lyon et al., 2010). External resistance also determines the quality of energy available for the growth and maintenance of microorganisms: bacteria obtain sufficient energy only at lower external resistances, which are associated with lower anode potentials and higher electron fluxes (Aelterman et al., 2008). In addition, our results suggested that a neutral pH condition yielded better performance across the majority of indices compared to acidic or alkaline conditions. This finding is consistent with many previous studies (Tremouli et al., 2017), even though some researchers have reported better power generation performance under acidic or alkaline conditions than at neutral pH (Dekker et al., 2009; He et al., 2008). This is likely because a neutral pH is optimal for the majority of microbes, whereas microbial activities can be inhibited—or even halted—at pH levels that are too high or too low (Puig et al., 2010).

### Effects of substrate characteristics on the power generation performance of MFCs

Substrate characteristics were also key factors affecting the power generation performance of MFCs. We found that substrates with pre-treatment significantly improved power density, voltage, and power, which were in line with other studies (He et al., 2025; Li et al., 2024).

By increasing the biodegradability of substrates, pre-treatment can boost the microbial utilization efficiency of the inoculum. Substrates can generally be pre-treated using physical, chemical, or biological, or combined (two or more pre-treatments) methods to modify the chemical structure of organic matter and support electricity-producing bacteria (He et al., 2025; Oh et al., 2014). Pre-treatment increases the biodegradability of substrates, making it easier for them to break down organic matter and release more organic compounds, thereby improving power density, voltage, and power output (Ray and Ghangrekar, 2015). Pre-treatments can also enhance core metabolic pathways—including those involved in energy, carbohydrate, nucleotide, lipid, and amino acid metabolism—and change the metabolite components of biofilms, thereby improving the power generation performance of MFC (He et al., 2025). Furthermore, our results suggested that biological pre-treatment yielded the highest power density, while physical and chemical pre-treatment were more effective at improving voltage and coulombic efficiency. However, this finding contrasts with a recent meta-analysis that found no significant differences in power generation performance among pre-treatment methods (Zafar et al., 2022). This discrepancy may be explained by the gentler nature of biological pre-treatment, in contrast to physical and chemical approaches, which often involve crushing, high temperatures, and the use of chemical catalysts or additives (Oh et al., 2014; Cao et al., 2018). These harsher treatments can damage microbial structures, reducing the effectiveness of power density improvement. To achieve higher power density, biological pre-treatments often include microbial inoculation, aerobic fermentation, or anaerobic fermentation to prevent the formation of inhibitory substances in the MFCs. For example, Rajesh et al. (2015) found that methane production reduced MFC power output by depleting available substrates. To address this, he inoculated the marine diatom *Chaetoceros* using a biopreparation method, which reduced methanogenic activity by 60%. This led to an increase in power density and overall performance. The non-significant effect of substrate pre-treatment on coulombic efficiency may be due to the fact that the majority of substrates in our study were simple, and their coulombic efficiency may not be significantly impacted by pre-treatment. Current densities in the unpretreated group were consistently higher than those in the pre-treated group. This could be because biological pre-treatment overly degraded the substrates, producing short-chain fatty acids such as acetic acid, propionic acid, or phenolics that inhibit the proper functioning of the electron transport chain in electroactive bacteria. As a result, the treatment's current density may have been somewhat lower than before treatment (Hassan et al., 2012). Chemical pre-treatment may also leave behind residual substances—such as H^+^, Cl^−^, free radicals, or remnants of acid, alkali, or oxidant pre-treatment—that can improve power generation performance at optimal concentrations but may exhibit toxic effects at higher doses. These residues can directly harm electroactive bacteria or damage the structure of the biofilm (He et al., 2025).

Additionally, the use of carbon felt as the electrode material, which is prone to pore blockage, can reduce biofilm binding sites and lower current density. This issue is further exacerbated by excessive grinding, high temperature, high pressure, or other treatments that result in substrate particles that are too small. Therefore, it is important to carefully consider whether pre-treatment methods may negatively impact the substrate or the entire MFC system, potentially leading to reduced current density. In summary, pre-treatment generally enhances power generation performance, and biological pre-treatment showed greater improvements compared to other pre-treatment methods.

In addition, our results indicated that solid substrates produced higher power, voltage, and current densities compared to liquid and fluid substrates, while liquid substrates exhibited higher coulombic efficiencies. These findings are consistent with previous studies that found that solid substrates had higher electrical performance than liquid and fluid substrates (Touch et al., 2014; Zhao et al., 2017), although they contradict others that suggested that solid substrates limited mass transfer due to insufficient physical transport rates of reactants at the electrode interface or within the reaction system. This limitation may result in a lower electrochemical reaction rate than the theoretical maximum, thereby restricting the electrical performance of MFCs. To improve the stability of power production performance, complex substrates are typically pre-treated—physically, chemically, or biologically—to reduce particle size, break down complex polysaccharides into monosaccharides, and inhibit methane production. This supports the earlier discussion on the effectiveness of pre-treatment methods. The majority of studies have used liquid and fluid substrates (e.g., wastewater and sludge), while relatively few have explored the use of solid substrates. Therefore, future studies may need to further investigate whether solid substrates consistently perform better for power generation than liquid and fluid substrates.

## Conclusion

This study was based on 186 publications and 10,826 experimental cases from the China National Knowledge Infrastructure (CNKI) database, systematically revealing the electricity generation performance characteristics of MFCs and their influencing factors. The results indicated that the power density, voltage, and reaction duration of MFCs in China were significantly lower than those reported in other meta-analyses or review papers. However, both coulombic efficiency and temperature were higher than those of international studies. These differences may be attributed to the inclusion of data across the entire polarization curve stage in this study and the prevalent use of dual-chamber reactors in Chinese experiments. Although MFCs have shown promising potential for pollutant removal, their low electricity generation performance remains a limiting factor for large-scale applications. Therefore, this study examined the effects of various influencing factors on electricity generation performance. Regarding substrate characteristics, the use of solid substrates or applying biological pre-treatment generally improved the majority of electricity generation performance indices. In terms of MFC device configurations, dual-chamber reactors tended to show higher electricity generation performance than single-chamber reactors. To obtain higher voltage and power, selecting a larger cathode area or cathode chamber volume is recommended. To achieve higher power density, current density, and coulombic efficiency, a smaller cathode area or reaction chamber volume may be more effective. Regarding reaction conditions, it is essential to select appropriate pH and temperature values to optimize electricity generation performance. Additionally, attention should be paid to reaction duration to avoid excessive reaction duration, which could lead to substrate depletion and lower electricity generation performance indices. In practical applications, the electricity generation performance of MFCs cannot be determined by a single factor alone. Therefore, this study explored the interactive effects of substrate characteristics, MFC device characteristics, and reaction conditions on electricity generation performance. Ultimately, it was determined that external resistance and cathode chamber volume were the primary factors influencing electricity generation performance during MFC operation. In this study, the explanatory power of individual influencing factors on voltage was relatively low, suggesting that other unaccounted factors may be restricting the improvement of voltage. Additionally, many studies did not provide information on cathode chamber volume or cathode material area, indicating that these parameters may not have been fully recognized as performance-influencing factors. This lack of data also prevented normalization, which may have introduced slight deviations in the analysis. This study has several limitations, including the exclusive use of Chinese publications, the absence of information on anode and cathode materials, and insufficient data on coulombic efficiency and power, all of which may limit the broader applicability of the findings.

## Data Availability

The raw data supporting the conclusions of this article will be made available by the authors, without undue reservation.

## References

[B1] Abourached C. Catal T. Liu H. (2014). Efficacy of single-chamber microbial fuel cells for removal of cadmium and zinc with simultaneous electricity production. Water Res. 51, 228–233. 10.1016/j.watres.2013.10.062 24289949

[B2] Abubackar H. N. Biryol I. Ayol A. (2023). Yeast industry wastewater treatment with microbial fuel cells: Effect of electrode materials and reactor configurations. Int. J. Hydrogen Energy 48, 12424–12432. 10.1016/j.ijhydene.2022.05.277

[B3] Aelterman P. Versichele M. Marzorati M. Boon N. Verstraete W. (2008). Loading rate and external resistance control the electricity generation of microbial fuel cells with different three-dimensional anodes. Bioresour. Technol. 99, 8895–8902. 10.1016/j.biortech.2008.04.061 18524577

[B4] Agrahari R. Bayar B. Abubackar H. N. Giri B. S. Rene E. R. Rani R. (2022). Advances in the development of electrode materials for improving the reactor kinetics in microbial fuel cells. Chemosphere 290:133184. 10.1016/j.chemosphere.2021.133184 34890618

[B5] Aiswaria P. Mohamed S. N. Singaravelu D. L. Brindhadevi K. Pugazhendhi A. (2022). A review on graphene/graphene oxide supported electrodes for microbial fuel cell applications: challenges and prospects. Chemosphere 296:133983. 10.1016/j.chemosphere.2022.133983 35181417

[B6] Amin M. M. Arvin A. Feizi A. Dehdashti B. Torkian A. (2022). Meta-analysis of bioenergy recovery and anaerobic digestion in integrated systems of anaerobic digestion and microbial electrolysis cell. Biochem. Eng. J. 178:108301. 10.1016/j.bej.2021.108301

[B7] An J. Kim B. Jang J. K. Lee H. S. Chang I. S. (2014). New architecture for modulization of membraneless and single-chambered microbial fuel cell using a bipolar plate-electrode assembly (BEA). Biosens. Bioelectron. 59, 28–34. 10.1016/j.bios.2014.02.063 24690558

[B8] An J. Sim J. Feng Y. J. Lee H. S. (2016). Understanding energy loss in parallelly connected microbial fuel cells: non-Faradaic current. Bioresour. Technol. 203, 280–286. 10.1016/j.biortech.2015.12.033 26744801

[B9] Babanova S. Jones J. Phadke S. Lu M. Q. Angulo C. Garcia J.. (2020). Continuous flow, large-scale, microbial fuel cell system for the sustained treatment of swine waste. Water Environ. Res. 92, 60–72. 10.1002/wer.1183 31306532

[B10] Bird H. Heidrich E. S. Leicester D. D. Theodosiou P. (2022). Pilot-scale microbial fuel cells (MFCs): a meta-analysis study to inform full-scale design principles for optimum wastewater treatment. J. Clean. Prod. 346:131227. 10.1016/j.jclepro.2022.131227

[B11] Cao B. C. Zhao Z. P. Peng L. L. Shiu H. Y. Ding M. N. Song F.. (2021). Silver nanoparticles boost charge-extraction efficiency in Shewanella microbial fuel cells. Science 373, 1336–1340. 10.1126/science.abf3427 34529487

[B12] Cao J. S. He H. Y. Li C. Zhou R. H. Luo J. Y. Xu M. (2018). The effects of different pretreatment methods of sludge on microbial fuel cells. Environ. Sci. Res. 31, 1389–1398. 10.13198/j.issn.1001-6929.2018.05.17

[B13] Castellano-Hinojosa A. Gallardo-Altamirano M. J. Pozo C. González-Martínez A. González-López J. (2024). Hydraulic retention time drives changes in energy production and the anodic microbiome of a microbial fuel cell (MFC). J. Water Process Eng. 59:104966. 10.1016/j.jwpe.2024.104966

[B14] Chakma R. Hossain M. K. Paramasivam P. Bousbih R. Amami M. Toki G. F. I.. (2025). *Recent applications, challenges, and future prospects of microbial fuel* cells: a review. Glob. Chall. 9:2500004. 10.1002/gch2.202500004 40352631 PMC12065106

[B15] Chen W. Liu Z. Li Y. Xing X. Liao Q. Zhu X. (2021). Improved electricity generation, coulombic efficiency and microbial community structure of microbial fuel cells using sodium citrate as an effective additive. J. Power Sources 482:228947. 10.1016/j.jpowsour.2020.228947

[B16] Cheng S. Logan B. E. (2011). Increasing power generation for scaling up single-chamber air cathode microbial fuel cells. Bioresour. Technol. 102, 4468–4473. 10.1016/j.biortech.2010.12.104 21273062

[B17] Dekker A. Ter Heijne A. Saakes M. Hamelers H. V. M. Buisman C. J. N. (2009). Analysis and improvement of a scaled-up and stacked microbial fuel cell. Environ. Sci. Technol. 43, 9038–9042. 10.1021/es901939r 19943685

[B18] Di Lorenzo M. Thomson A. R. Schneider K. Cameron P. J. Ieropoulos I. (2014). A small-scale air-cathode microbial fuel cell for on-line monitoring of water quality. Biosens. Bioelectron. 62, 182–188. 10.1016/j.bios.2014.06.050 25005554

[B19] Dowdy F. R. Kawakita R. Lange M. Simmons C. W. (2018). Meta-analysis of microbial fuel cells using waste substrates. Appl. Biochem. Biotechnol. 185, 221–232. 10.1007/s12010-017-2652-8 29124654

[B20] Du Z. Li H. Gu T. (2007). A state of the art review on microbial fuel cells: a promising technology for wastewater treatment and bioenergy. Biotechnol. Adv. 25, 464–482. 10.1016/j.biotechadv.2007.05.004 17582720

[B21] Elmekawy A. Hegab H. M. Losic D. Saint C. P. Pant D. (2017). Applications of graphene in microbial fuel cells: the gap between promise and reality. Renew. Sustain. Energy Rev. 72, 1389–1403. 10.1016/j.rser.2016.10.044

[B22] Flimban S. G. A. Ismail I. M. I. Kim T. Oh S. E. (2019). Overview of recent advancements in the microbial fuel cell from fundamentals to applications: design, major elements, and scalability. Energies 12:3390. 10.3390/en12173390

[B23] Fouchecour F. Larzilliere V. Bouchez T. Moscoviz R. (2022). Systematic and quantitative analysis of two decades of anodic wastewater treatment in bioelectrochemical reactors. Water Res. 214:118142. 10.1016/j.watres.2022.118142 35217490

[B24] Fu Q. Fukushima N. Maeda H. Sato K. Kobayashi H. (2015). Bioelectrochemical analysis of a hyperthermophilic microbial fuel cell generating electricity at temperatures above 80°C. Biosci. Biotechnol. Biochem. 79, 1200–1206. 10.1080/09168451.2015.1015952 25747034

[B25] Gao H. J. Wang Z. Yu Y. L. Lv C. C. Li W. Y. Guo J. Y.. (2024). Study on nitrogen degradation and electricity generation performance of microbial fuel cell constructed wetland for sewage. J. Hydroecol. 45, 192–203. 10.15928/j.1674-3075.202402230051

[B26] Ge X. Cao X. Song X. Wang Y. Si Z. Zhao Y.. (2020). Bioenergy generation and simultaneous nitrate and phosphorus removal in a pyrite-based constructed wetland-microbial fuel cell. Bioresour. Technol. 296:122350. 10.1016/j.biortech.2019.122350 31744666

[B27] Ge Z. He Z. (2016). Long-term performance of a 200 liter modularized microbial fuel cell system treating municipal wastewater: treatment, energy, and cost. Environ. Sci. Water Res. Technol. 2, 274–281. 10.1039/C6EW00020G

[B28] Green F. Von Kursk O. B. Muttitt G. Pye S. (2024). No new fossil fuel projects: the norm we need. Science 384, 954–957. 10.1126/science.adn6533 38815017

[B29] Grondin F. Perrier M. Tartakovsky B. (2012). Microbial fuel cell operation with intermittent connection of the electrical load. J. Power Sources 208, 18–23. 10.1016/j.jpowsour.2012.02.010

[B30] Gupta S. Patro A. Mittal Y. Dwivedi S. Saket P. Panja R.. (2023). The race between classical microbial fuel cells, sediment-microbial fuel cells, plant-microbial fuel cells, and constructed wetlands-microbial fuel cells: applications and technology readiness level. Sci. Total Environ. 879:162757. 10.1016/j.scitotenv.2023.162757 36931518

[B31] Haavisto J. M. Kokko M. E. Lay C.-H. Puhakka J. A. (2017). Effect of hydraulic retention time on continuous electricity production from xylose in up-flow microbial fuel cell. Int. J. Hydrogen Energy 42, 27494–27501. 10.1016/j.ijhydene.2017.05.068

[B32] Hassan S. H. A. Kim Y. S. Oh S. E. (2012). Power generation from cellulose using mixed and pure cultures of cellulose-degrading bacteria in a microbial fuel cell. Enzyme Microb. Technol. 51, 269–273. 10.1016/j.enzmictec.2012.07.008 22975124

[B33] He D. Nong Y. He Y. Luo Y. Li C. Gao J.. (2025). Effect of pre-chlorination on bioelectricity production and stabilization of excess sludge by microbial fuel cell. Water Res. 281:123564. 10.1016/j.watres.2025.123564 40184708

[B34] He Z. Huang Y. Manohar A. K. Mansfeld F. (2008). Effect of electrolyte pH on the rate of the anodic and cathodic reactions in an air-cathode microbial fuel cell. Bioelectrochemistry 74, 78–82. 10.1016/j.bioelechem.2008.07.007 18774345

[B35] Hiegemann H. Herzer D. Nettmann E. Lübken M. Schulte P. Schmelz K. G.. (2016). An integrated 45 L pilot microbial fuel cell system at a full-scale wastewater treatment plant. Bioresour. Technol. 218, 115–122. 10.1016/j.biortech.2016.06.052 27351707

[B36] Hindatu Y. Annuar M. S. M. Gumel A. M. (2017). Mini-review: anode modification for improved performance of microbial fuel cell. Renew. Sustain. Energy Rev. 73, 236–248. 10.1016/j.rser.2017.01.138

[B37] Hou H. Li L. De Figueiredo P. Han A. (2011). Air-cathode microbial fuel cell array: a device for identifying and characterizing electrochemically active microbes. Biosens. Bioelectron. 26, 2680–2684. 10.1016/j.bios.2010.06.037 20655725

[B38] Hu J. L. Dai J. H. Sun Z. T. Shi G. C. Zhang J. F. (2020). Research on sediment microbial fuel cell in the removal of sludge pollution. J. Waterw. Harb. 41, 725–730.

[B39] Jalili P. Ala A. Nazari P. Jalili B. Ganji D. D. (2024). A comprehensive review of microbial fuel cells considering materials, methods, structures, and microorganisms. Heliyon 10:e25439. 10.1016/j.heliyon.2024.e25439 38371992 PMC10873675

[B40] Jia B. Li X. M. Liu Z. H. Yang Q. Liao D. X. Zeng G. M.. (2008). Research and comparison of two-chamber and single-chamber microbial fuel cells. Environ. Pollut. Control 74–77+82. 10.3969/j.issn.1001-3865.2008.05.020

[B41] Jia Q. B. Wei L. L. Han H. L. Shen J. Q. (2014). Factors that influence the performance of two-chamber microbial fuel cell. Int. J. Hydrogen Energy 39, 13687–13693. 10.1016/j.ijhydene.2014.04.023

[B42] Khan N. Khan M. D. Nizami A. S. Rehan M. Shaida A. Ahmad A.. (2018). Energy generation through bioelectrochemical degradation of pentachlorophenol in microbial fuel cell. RSC Adv. 8, 20726–20736. 10.1039/C8RA01643G 35542361 PMC9080799

[B43] Krieg T. Mayer F. Sell D. Holtmann D. (2019). Insights into the applicability of microbial fuel cells in wastewater treatment plants for a sustainable generation of electricity. Environ. Technol. 40, 1101–1109. 10.1080/09593330.2017.1401668 29105566

[B44] Lee Y. Y. Kim T. G. Cho K. S. (2016). Characterization of the COD removal, electricity generation, and bacterial communities in microbial fuel cells treating molasses wastewater. J. Environ. Sci. Health A. Tox. Hazard Subst. Environ. Eng. 51, 1131–1138. 10.1080/10934529.2016.1199926 27428492

[B45] Li H. Cao H. Li T. He Z. Zhao J. Zhang Y.. (2023). Biofilm electrode reactor coupled manganese ore substrate up-flow microbial fuel cell-constructed wetland system: High removal efficiencies of antibiotic, zinc (II), and the corresponding antibiotic resistance genes. J. Hazard. Mater. 460:132394. 10.1016/j.jhazmat.2023.132394 37657329

[B46] Li L. H. Sun Y. M. Yuan Z. H. Kong X. Y. Li Y. (2013). Effect of temperature change on power generation of microbial fuel cell. Environ. Technol. 34, 1929–1934. 10.1080/09593330.2013.828101 24350446

[B47] Li S. Y. Wang F. Yang X. Z. Wang Y. L. Zhang D. L. Yang F. L.. (2024). Effects of aeration coupled with microbial fuel cell on nitrogen removal and electricity production for biogas slurry. J. Environ. Chem. Eng. 12:113761. 10.1016/j.jece.2024.113761

[B48] Li X. J. Wang X. Weng L. P. Zhou Q. X. Li Y. T. (2017). Microbial fuel cells for organic-contaminated soil remedial applications: a review. Energy Technol. 5, 1156–1164. 10.1002/ente.201600674

[B49] Lin C. Y. Liang H. R. Yang X. J. Zhan J. J. Yang Q. (2024). Voltage recovery from frozen microbial fuel cells in the laboratory and outdoor field reactors. Sci. Total Environ. 942:173751. 10.1016/j.scitotenv.2024.173751 38839000

[B50] Liu H. Cheng S. Logan B. E. (2005). Power generation in fed-batch microbial fuel cells as a function of ionic strength, temperature, and reactor configuration. Environ. Sci. Technol. 39, 5488–5493. 10.1021/es050316c 16082985

[B51] Liu L. H. Tsyganova O. Lee D. J. Su A. Chang J. S. Wang A. J.. (2012). Anodic biofilm in single-chamber microbial fuel cells cultivated under different temperatures. Int. J. Hydrogen Energy 37, 15792–15800. 10.1016/j.ijhydene.2012.03.084

[B52] Logan B. Cheng S. Watson V. Estadt G. (2007). Graphite fiber brush anodes for increased power production in air-cathode microbial fuel cells. Environ. Sci. Technol. 41, 3341–3346. 10.1021/es062644y 17539547

[B53] Logan B. E. Rabaey K. (2012). Conversion of wastes into bioelectricity and chemicals by using microbial electrochemical technologies. Science 337, 686–690. 10.1126/science.1217412 22879507

[B54] Logan B. E. Rossi R. Ragab A. A. Saikaly P. E. (2019). Electroactive microorganisms in bioelectrochemical systems. Nat. Rev. Microbiol. 17, 307–319. 10.1038/s41579-019-0173-x 30846876

[B55] Lu M. Chen S. Babanova S. Phadke S. Salvacion M. Mirhosseini A.. (2017). Long-term performance of a 20-L continuous flow microbial fuel cell for treatment of brewery wastewater. J. Power Sources 356, 274–287. 10.1016/j.jpowsour.2017.03.132

[B56] Lyon D. Y. Buret F. Vogel T. M. Monier J.-M. (2010). Is resistance futile? Changing external resistance does not improve microbial fuel cell performance. Bioelectrochemistry 78, 2–7. 10.1016/j.bioelechem.2009.09.001 19783225

[B57] Mahmoudi A. Mousavi S. A. Darvishi P. (2024). Performance and recent development in sewage sludge-to-bioenergy using microbial fuel cells: a comprehensive review. Int. J. Hydrogen Energy 50, 1432–1455. 10.1016/j.ijhydene.2023.10.338

[B58] Margaria V. Tommasi T. Pentassuglia S. Agostino V. Sacco A. Armato C.. (2017). Effects of pH variations on anodic marine consortia in a dual chamber microbial fuel cell. Int. J. Hydrogen Energy 42, 1820–1829. 10.1016/j.ijhydene.2016.07.250

[B59] Mei X. Xing D. Yang Y. Liu Q. Zhou H. Guo C.. (2017). Adaptation of microbial community of the anode biofilm in microbial fuel cells to temperature. Bioelectrochemistry 117, 29–33. 10.1016/j.bioelechem.2017.04.005 28575837

[B60] Nimje V. R. Chen C.-C. Chen H.-R. Chen C.-Y. Tseng M.-J. Cheng K.-C.. (2012). A single-chamber microbial fuel cell without an air *cathode*. Int. J. Mol. Sci. 13, 3933–3948. 10.3390/ijms13033933 22489190 PMC3317750

[B61] Obata O. Salar-Garcia M. J. Greenman J. Kurt H. Chandran K. Ieropoulos I. (2020). Development of efficient electroactive biofilm in urine-fed microbial fuel cell cascades for bioelectricity generation. J. Environ. Manage. 258:109992. 10.1016/j.jenvman.2019.109992 31929046 PMC7001104

[B62] Oh S. E. Yoon J. Y. Gurung A. Kim D. J. (2014). Evaluation of electricity generation from ultrasonic and heat/alkaline pretreatment of different sludge types using microbial fuel cells. Bioresour. Technol. 165, 21–26. 10.1016/j.biortech.2014.03.018 24684816

[B63] Olabi A. G. Wilberforce T. Sayed E. T. Elsaid K. Rezk H. Abdelkareem M. A. (2020). Recent progress of graphene based nanomaterials in bioelectrochemical systems. Sci. Total Environ. 749:141225. 10.1016/j.scitotenv.2020.141225 32814206

[B64] Opoku P. A. Guang L. Huang J. Y. Norgbey E. (2022). Impact of wastewater volume on cathode environment of the multi-anode shared cathode and standard single anode/cathode microbial fuel cells. Chem. Pap. 76, 6309–6321. 10.1007/s11696-022-02316-8

[B65] Pandey H. Pandey R. R. Andola A. Prakash A. Pandey R. K. (2024). Emerging frontiers in microbial fuel cell technology for sustainable energy generation. J. Solid State Electrochem. 10.1007/s10008-024-06167-z

[B66] Pasupuleti S. B. Srikanth S. Mohan S. V. Pant D. (2015). Continuous mode operation of microbial fuel cell (MFC) stack with dual gas diffusion cathode design for the treatment of dark fermentation effluent. Int. J. Hydrogen Energy 40, 12424–12435. 10.1016/j.ijhydene.2015.07.049

[B67] Prasad J. Tripathi R. K. (2022). Review on improving microbial fuel cell power management systems for consumer applications. Energy Rep. 8, 10418–10433. 10.1016/j.egyr.2022.08.192

[B68] Puig S. Serra M. Coma M. Cabre M. Balaguer M. D. Colprim J. (2010). Effect of pH on nutrient dynamics and electricity production using microbial fuel cells. Bioresour. Technol. 101, 9594–9599. 10.1016/j.biortech.2010.07.082 20702091

[B69] Qian F. Baum M. Gu Q. Morse D. E. (2009). A 1.5 microL microbial fuel cell for on-chip bioelectricity generation. Lab Chip 9, 3076–3081. 10.1039/b910586g 19823722

[B70] Qin G. Zhang X. B. Yu R. D. Chen J. Len X. An S. Q. (2021). Research on the removal of typical PPCPs by constructed wetland microbial fuel cell. Wetl. Sci. Manag. 17, 8–11+17.

[B71] Rajesh P. P. Jadhav D. A. Ghangrekar M. M. (2015). Improving performance of microbial fuel cell while controlling methanogenesis by chaetoceros pretreatment of anodic inoculum. Bioresour. Technol. 180, 66–71. 10.1016/j.biortech.2014.12.095 25590424

[B72] Ray S. G. Ghangrekar M. M. (2015). Enhancing organic matter removal, biopolymer recovery and electricity generation from distillery wastewater by combining fungal fermentation and microbial fuel cell. Bioresour. Technol. 176, 8–14. 10.1016/j.biortech.2014.10.158 25460978

[B73] Shi X. Y. Yang D. W. Li S. S. Yu K. D. Yan W. Xu H. (2025). Research progress on coupling and stacking systems to enhance power generation performance of microbial fuel cell. J. Environ. Sci. 154, 784–804. 10.1016/j.jes.2024.10.003 40049916

[B74] Shindell D. Smith C. J. (2019). Climate and air-quality benefits of a realistic phase-out of fossil fuels. Nature, 573, 408–411. 10.1038/s41586-019-1554-z 31534245

[B75] Slate A. J. Whitehead K. A. Brownson D. A. C. Banks C. E. (2019). Microbial fuel cells: an overview of current technology. Renew. Sustain. Energy Rev. 101, 60–81. 10.1016/j.rser.2018.09.044

[B76] Sonawane J. M. Mahadevan R. Pandey A. Greener J. (2022). Recent progress in microbial fuel cells using substrates from diverse sources. Heliyon 8:e12353. 10.1016/j.heliyon.2022.e12353 36582703 PMC9792797

[B77] Sonawane J. M. Yadav A. Ghosh P. C. Adeloju S. B. (2017). Recent advances in the development and utilization of modern anode materials for high performance microbial fuel cells. Biosens. Bioelectron. 90, 558–576. 10.1016/j.bios.2016.10.014 27825877

[B78] Song X. R. Jo C. Han L. J. Zhou M. H. (2022). Recent advance in microbial fuel cell reactor configuration and coupling technologies for removal of antibiotic pollutants. Curr. Opin. Electrochem. 31:100833. 10.1016/j.coelec.2021.100833

[B79] Song Y. An J. Chae K. J. (2017). Effect of temperature variation on the performance of microbial fuel cells. Energy Technol. 5, 2163–2167. 10.1002/ente.201700277

[B80] Sorgato A. C. Jeremias T. C. Lobo F. L. Lapolli F. R. (2023). Microbial fuel cell: Interplay of energy production, wastewater treatment, toxicity assessment with hydraulic retention time. Environ. Res. 231:116159. 10.1016/j.envres.2023.116159 37211179

[B81] Sun Z. T. Zhang J. F. Zhang Q. H. (2022). Sediment microbial fuel cell for aquaculture water remediation research. Environ. Pollut. Prev. Control 44, 841−845+853. 10.15985/j.cnki.1001-3865.2022.07.001

[B82] Tao M. Jing Z. Shen Y. Cao S. Li Y.-Y. (2025). Nitrogen and phosphorus removal in microbial fuel cell-constructed wetland integrated with layered double hydroxides coated filter for treating low carbon wastewater. J. Water Process Eng. 70:106907. 10.1016/j.jwpe.2024.106907

[B83] Tee P. F. Abdullah M. O. Tan I. A. W. Amin M. A. M. Nolasco-Hipolito C. Bujang K. (2018). Bio-energy generation in an affordable, single-chamber microbial fuel cell integrated with adsorption hybrid system: effects of temperature and comparison study. Environ. Technol. 39, 1081–1088. 10.1080/09593330.2017.1320433 28417676

[B84] Touch N. Hibino T. Nagatsu Y. Tachiuchi K. (2014). Characteristics of electricity generation and biodegradation in tidal river sludge-used microbial fuel cells. Bioresour. Technol. 158, 225–230. 10.1016/j.biortech.2014.02.035 24607458

[B85] Tremouli A. Martinos M. Lyberatos G. (2017). The effects of salinity, pH and temperature on the performance of a microbial fuel cell. Waste Biomass Valorization 8, 2037–2043. 10.1007/s12649-016-9712-0

[B86] Uddin M. J. Jeong Y.-K. Lee W. (2021). Microbial fuel cells for bioelectricity generation through reduction of hexavalent chromium in wastewater: a review. Int. J. Hydrogen Energy 46, 11458–11481. 10.1016/j.ijhydene.2020.06.134

[B87] Vogt E. T. C. Weckhuysen B. M. (2024). The refinery of the future. Nature 629, 295–306. 10.1038/s41586-024-07322-2 38720037

[B88] Wang J. F. Song X. S. Wang Y. H. Abayneh B. Li Y. H. Yan D. H.. (2016). Nitrate removal and bioenergy production in constructed wetland coupled with microbial fuel cell: Establishment of electrochemically active bacteria community on anode. Bioresour. Technol. 221, 358–365. 10.1016/j.biortech.2016.09.054 27658173

[B89] Wang Y. Wang Z. Kong X. Song Y. Tian Y. Lin J. (2024). Capacitive and biocompatibility composite anode material for enhanced renewable energy conversion for MFCs. Fuel 376:132736. 10.1016/j.fuel.2024.132736

[B90] Wei L. Li Z. Y. Hong T. Q. Tang Y. M. Ge Y. Ji D. D.. (2024). Insight into electricity production performance from dried waste activated sludge in dual chamber microbial fuel cells: Influencing factors, neural network modelling and microbial community analysis. J. Environ. Chem. Eng. 12:113477. 10.1016/j.jece.2024.113477

[B91] Wei L. L. Han H. L. Shen J. Q. (2013). Effects of temperature and ferrous sulfate concentrations on the performance of microbial fuel cell. Int. J. Hydrogen Energy 38, 11110–11116. 10.1016/j.ijhydene.2013.01.019

[B92] Xu L. Zhao Y. Q. Doherty L. Hu Y. S. Hao X. D. (2016). The integrated processes for wastewater treatment based on the principle of microbial fuel cells: a review. Crit. Rev. Environ. Sci. Technol. 46, 60–91. 10.1080/10643389.2015.1061884

[B93] Yu J. You J. Lens P. N. L. Lu L. He Y. Ji Z.. (2023). Biofilm metagenomic characteristics behind high coulombic efficiency for propanethiol deodorization in two-phase partitioning microbial fuel cell. Water Res. 246:120677. 10.1016/j.watres.2023.120677 37827037

[B94] Zadeh P. G. Rezania S. Fattahi M. Dang P. Y. Vasseghian Y. Aminabhavi T. M. (2024). Recent advances in microbial fuel cell technology for energy generation from wastewater sources. Process Saf. Environ. Prot. 189, 425–439. 10.1016/j.psep.2024.06.077

[B95] Zafar H. Ishaq S. Peleato N. Roberts D. (2022). Meta-analysis of operational performance and response metrics of microbial fuel cells (MFCs) fed with complex food waste. J. Environ. Manage. 315:115152. 10.1016/j.jenvman.2022.115152 35525044

[B96] Zhang J. F. Shi G. C. Sun Z. T. Hu J. L. (2022). The application of sediment microbial fuel cell (SMFC) in electricity generation and organic matter removal in estuarine sediment. J. Tianjin Univ. 55, 85–89. 10.11784/tdxbz202101008

[B97] Zhang L. B. Li C. Ding L. L.Q R.H. (2010). The influence of temperature and pH on the electricity generation of microbial fuel cells. Environ. Pollut. Control 32, 62–66. 10.15985/j.cnki.1001-3865.2010.04.020

[B98] Zhang Q. G. Hu J. J. Lee D. J. (2016). Microbial fuel cells as pollutant treatment units: research updates. Bioresour. Technol. 217, 121–128. 10.1016/j.biortech.2016.02.006 26906446

[B99] Zhang X. He W. Ren L. Stager J. Evans P. J. Logan B. E. (2015). COD removal characteristics in air-cathode microbial fuel cells. Bioresour. Technol. 176, 23–31. 10.1016/j.biortech.2014.11.001 25460980

[B100] Zhang X. Xiao Y. Zhou Q. H. Wu Z. B. (2017). Research progress of electricity-producing microorganisms in microbial fuel cells. Biotechnol. Bull. 33, 64–73. 10.13560/j.cnki.biotech.bull.1985.2017-0222

[B101] Zhao Q. Yu H. Zhang W. Kabutey F. T. Jiang J. Zhang Y.. (2017). Microbial fuel cell with high content solid wastes as substrates:a review. Front. Environ. Sci. Eng. 11, 25–41. 10.1007/s11783-017-0918-6

[B102] Zhao Y. Bo X. Ma Y. Wang J. W. Wang Y. P. Li T.. (2014). The operational characteristics of microbial fuel cells at different temperatures. Chem. Ind. Eng. Prog. 33, 629–633+650. 10.3969/j.issn.1000-6613.2014.03.018

