# Supplementary material for: Characteristics and driving factors of power generation performance in microbial fuel cells: an analysis based on the CNKI database
Source: Front Microbiol. 2025 Jun 13;16:1620539. doi: 10.3389/fmicb.2025.1620539 (PMC12202401; doi:10.3389/fmicb.2025.1620539)
Supplement: Supplementary file 1 [file Table_1.docx]

Table S1 Data transformation in this study.

| Factors | Transformation method | Number of cases after screening |
| --- | --- | --- |
| Anode surface area | Y=ln (1+X) | 7028 |
| Cathode surface area | Y=ln (1+X) | 6501 |
| Anode chamber volume | Y=ln (1+X) | 5944 |
| Cathode chamber volume | Y=ln (1+X) | 5309 |
| Total volume of reaction chamber | Y=ln (1+X) | 7292 |
| Battery start-up time | Y=ln (1+X) | 2047 |
| Reaction duration | Y=ln (1+X） | 5176 |
| External resistance | Y=lnX | 5786 |
| pH | Y=X^2^ | 2486 |
| Cathode pH | Y=lnX | 1014 |
| Temperature | Y=lnX | 4436 |
| Internal Resistance (Ω) | Y=ln (1+X) | 1345 |
| Voltage (mV) | Y=ln (1+X) | 7401 |
| Power (W) | Y=lnX | 177 |
| Power density (W/m^3^) | Y=ln (1+X) | 1096 |
| Power density (mW/m^2^) | Y=ln (1+X) | 3165 |
| Coulomb efficiency (%) | Y=X^1/2^ | 337 |
| Current density (mA/m^2^) | Y=ln (1+X) | 3071 |
| Current density (A/m^3^) | Y=ln (1+X) | 1019 |

Table S2 Data transformation of this study in comparison with other meta-analysis or review papers.

| Factors | Transformation method | Number of cases after screening |
| --- | --- | --- |
| Operating temperature | - | 4517 |
| Power density (mW/m^2^) | Y=ln (1+X) | 3311 |
| Power density (W/m^3^) | Y=ln (1+X) | 1168 |
| Voltage (mV) | Y=ln (1+X) | 7543 |
| Coulomb efficiency (%) | Y=ln (1+X) | 454 |
| Reaction duration | Y=ln (1+X) | 5212 |

Table S3 The individual and interaction effects of different influencing factors on the power generation performance indices.

| Parameters | Voltage (mV) | Power  (W) | Power density (mW/m^2^) | Power density (W/m^3^) | coulombic efficiency (%) | Current density (mA/m^2^) | Current density (A/m^3^) |
| --- | --- | --- | --- | --- | --- | --- | --- |
| CMA |  |  | **< 0.001** | **< 0.001** | **< 0.001** | **< 0.001** | **0.042** |
| P_1_ |  |  | 0.065 | **< 0.001** | 0.496 | **< 0.001** | **< 0.001** |
| CMA×P_1_ |  |  | **< 0.001** | **< 0.001** | 0.093 | 0.125 | **< 0.001** |
| CMA |  |  | **< 0.001** | **< 0.001** | **<0.001** | **< 0.001** | **< 0.001** |
| P_2_ |  |  | **< 0.001** | **< 0.001** | **<0.001** | 0.849 | **< 0.001** |
| CMA×P_2_ |  |  | **< 0.001** | **< 0.001** | **<0.001** | 0.799 | **< 0.001** |
| CMA |  |  | **< 0.001** | **< 0.001** | **<0.001** | **< 0.001** | **< 0.001** |
| P_3_ |  |  | **< 0.001** | **< 0.001** | **<0.001** | 0.434 | **< 0.001** |
| CMA×P_3_ |  |  | **< 0.001** | **0.003** | **<0.001** | **0.039** | **< 0.001** |
| CCV | **< 0.001** |  | **< 0.001** | 0.119 |  | **< 0.001** | **< 0.001** |
| P_1_ | **0.026** |  | **< 0.001** | **< 0.001** |  | **0.001** | **0.034** |
| CCV×P_1_ | **< 0.001** |  | **< 0.001** | **< 0.001** |  | **< 0.001** | **0.001** |
| CCV |  |  |  | **0.001** |  | **< 0.001** |  |
| P_2_ |  |  |  | 0.146 |  | **< 0.001** |  |
| CCV×P_2_ |  |  |  | 0.053 |  | 0.182 |  |
| CCV |  |  | **< 0.001** | **0.047** | **<0.001** | **< 0.001** | **< 0.001** |
| P_3_ |  |  | **< 0.001** | **< 0.001** | **<0.001** | **< 0.001** | **< 0.001** |
| CCV×P_3_ |  |  | **< 0.001** | **< 0.001** | **<0.001** | **0.019** | **< 0.001** |
| ER | **< 0.001** | **<0.001** | 0.090 |  | 0.291 | **< 0.001** | 0.856 |
| P_1_ | **< 0.001** | **<0.001** | **< 0.001** |  | **<0.001** | **< 0.001** | **< 0.001** |
| ER×P_1_ | **< 0.001** | **<0.001** | **< 0.001** |  | **<0.001** | **< 0.001** | **< 0.001** |
| ER | **< 0.001** |  | 0.852 |  |  | **< 0.001** | **< 0.001** |
| P_2_ | **0.001** |  | **< 0.001** |  |  | **< 0.001** | **< 0.001** |
| ER×P_2_ | **< 0.001** |  | **< 0.001** |  |  | **< 0.001** | **< 0.001** |
| ER | **< 0.001** | **0.003** | 0.151 |  | 0.486 | **< 0.001** |  |
| P_3_ | 0.343 | **0.028** | **< 0.001** |  | 0.315 | **< 0.001** |  |
| ER×P_3_ | 0.542 | **0.035** | **< 0.001** |  | 0.263 | **< 0.001** |  |
| RD | **< 0.001** |  | **< 0.001** | **< 0.001** |  | **< 0.001** | **< 0.001** |
| P_1_ | 0.777 |  | **< 0.001** | **< 0.001** |  | **< 0.001** | **< 0.001** |
| RD×P_1_ | **0.023** |  | **< 0.001** | **< 0.001** |  | **0.014** | **< 0.001** |
| RD | **< 0.001** |  | **< 0.001** | **< 0.001** |  | 0.588 | **< 0.001** |
| P_2_ | **< 0.001** |  | **0.002** | **0.032** |  | **0.004** | 0.304 |
| RD×P_2_ | **< 0.001** |  | **0.017** | **< 0.001** |  | **0.004** | 0.586 |
| RD | **0.001** |  | 0.859 | **< 0.001** |  | 0.062 | **< 0.001** |
| P_3_ | **0.043** |  | **< 0.001** | **< 0.001** |  | 0.552 | **< 0.001** |
| RD×P_3_ | **0.019** |  | **< 0.001** | **< 0.001** |  | **0.002** | **< 0.001** |
| BIR |  |  |  | **0.001** |  |  |  |
| P_1_ |  |  |  | 0.594 |  |  |  |
| BIR×P_1_ |  |  |  | 0.560 |  |  |  |
| BIR |  |  |  | **0.024** |  |  |  |
| P_2_ |  |  |  | **< 0.001** |  |  |  |
| BIR×P_2_ |  |  |  | **< 0.001** |  |  |  |
| BIR |  |  |  | **< 0.001** |  |  |  |
| P_3_ |  |  |  | 0.379 |  |  |  |
| BIR×P_3_ |  |  |  | 0.339 |  |  |  |
| BST |  |  |  | **< 0.001** |  |  |  |
| P_1_ |  |  |  | **< 0.001** |  |  |  |
| BST×P_1_ |  |  |  | **< 0.001** |  |  |  |

Note: P_1_ means whether to perform pretreatment, P_2_ means Reaction chamber type, P_3_ means Substrate type, CMA means cathode material area, CCV means cathode chamber volume, ER means external resistance, RD means reaction duration, BIR means battery internal resistance, BST means battery startup time

|  | Voltage  (mV) | Power  (W) | Power Density  (mW/m^2^) | Power Density  (W/m^3^) | Coulombic efficiency  (%) | Current density  (mA/m^2^) | Current density  (A/m^3^) |
| --- | --- | --- | --- | --- | --- | --- | --- |
| Anode material area  (cm^2^) | 0.065 (4615)^***^ | -0.120 (32)^ns^ | -0.282 (2334)^***^ | 0.251 (609)^***^ | -0.507 (199)^***^ | -0.448 (2097)^***^ | -0.243 (510)^***^ |
| Cathode material area  (cm^2^) | 0.006 (4375)^ns^ | 0.313 (41)^*^ | -0.284 (2280)^***^ | 0.232 (561)^***^ | -0.529 (196)^***^ | -0.446 (2038)^***^ | 0.179 (496)^***^ |
| Anode chamber volume  (mL) | 0.068 (3968)^***^ | 0.955 (44)^***^ | -0.277 (1651)^***^ | 0.295 (865)^***^ | -0.658 (189)^***^ | -0.341 (1649)^***^ | -0.103 (614)^**^ |
| Cathode chamber volume  (mL) | 0.069 (3511)^***^ | 0.493 (51)^***^ | -0.325 (1428)^***^ | 0.146 (725)^***^ | -0.656 (185)^***^ | -0.403 (1517)^***^ | -0.270 (369)^***^ |
| Reaction chamber volume  (mL) | -0.093 (4728)^***^ | 0.416 (109)^***^ | -0.410 (2069)^***^ | -0.169 (940)^***^ | -0.097 (159)^ns^ | -0.394 (2110)^***^ | -0.421 (873)^***^ |
| Battery start-up time (d) | -0.023 (1533)^ns^ |  | -0.063 (419)^ns^ | 0.000 (292)^ns^ | 0.659 (174)^***^ | 0.115 (407)^**^ | -0.040 (106)^ns^ |
| Reaction duration (h) | 0.104 (4029)^***^ | -0.771 (78)^***^ | -0.212 (568)^***^ | 0.767 (188)^***^ | 0.013 (82)^ns^ | -0.198 (626)^***^ | 0.623 (85)^***^ |
| External resistance (Ω) | 0.248 (4356)^***^ | -0.644 (54)^***^ | -0.028 (1608)^ns^ | 0.002 (491)^ns^ | -0.196 (293)^**^ | 0.305 (1470)^***^ | -0.271 (540)^***^ |
| pH | 0.011 (1856)^***^ |  | 0.523 (487)^***^ | -0.116 (313)^*^ | 0.851 (80)^***^ | 0.309 (805)^***^ | -0.070 (271)^ns^ |
| Temperature (℃) | 0.020 (2944)^ns^ | -0.100 (60)^ns^ | -0.080 (1384)^**^ | 0.111 (672)^**^ | 0.116 (157)^ns^ | -0.081 (1409)^**^ | 0.009 (755)^ns^ |
| Internal Resistance (Ω) | 0.028 (836)^ns^ |  | -0.170 (701)^***^ | -0.385 (241)^***^ | 0.185 (115)^*^ | 0.258 (736)^***^ | -0.286 (57)^**^ |

Table S4 Results of correlation analyses between MFC power generation performance indices and device configurations and reaction conditions.

Note: Data are shown as correlation coeffieient and number of cases (in parentheses). * P<0.05, ** P < 0.01, *** P < 0.001, ns not significant.

Appendix Ⅰ: References used in the meta-analysis to build the database (in Chinese).

1. Fan P., Song T., Qin B., et.al. Effects of different domestication methods on the power generation performance of microbial fuel cells with phenol as the substrate. Chinese Journal of Environmental Engineering. 2012, 6(11): 3867-3872.
2. Xiao P., Song T., Wu X., et.al. Effects of stainless-steel electrodeposited carbon nanotubes as electrodes on electricity generation in sedimentary microbial fuel cells. Renewable Energy. 2012, 30(12): 52-55.
3. Xiao L., Wang Y., Lin H., et.al. Preparation of stainless-steel fiber mat modified material and its application in microbial fuel cells. Chinese Journal of Environmental Engineering. 2023, 17(01): 288-298.
4. Dai H., Yang H., Liu X., et.al. Study on the power generation performance of stainless-steel mesh cathode microbial fuel cells. Electrochemistry. 2016, 22(01): 75-80.
5. Wang J., Xia X., Chen S., et.al. Experimental study on nitrate wastewater treatment by two types of microbial fuel cells. Acta Scientiae Circumologica Sinica. 2011, 31(02): 254-259.
6. Cheng J., Sun Q., Hu S., et.al. Study on nitrate degradation by two types of cathode bacterial communities in microbial fuel cells. Journal of Hefei University of Technology (Natural Science Edition). 2014, 37(10): 1168-1172.
7. Wang J., Li C., Tan Q. Study on the treatment of organic wastewater by single-chamber microbial fuel cell with lead dioxide cathode. Water Treatment Technology. 2009, 35(09): 84-86.
8. Huang Y., Lu Y., Huang Y., et.al. Study on the isolation, screening, identification and culture conditions of electric-producing Marine lipase-producing bacteria. Biotechnology Bulletin. 2020, 36(12): 91-97.
9. Li F., Zhou Q., Li B. The effects of electrically-producing microbiota and electron acceptors on microbial fuel cell performance. Chinese Journal of Applied Ecology. 2009, 20(12): 3070-3074.
10. Qin G., Chen J., Yu R., et.al. Study on enhanced purification and electricity generation of high carbon and nitrogen wastewater by constructed wet-microbial fuel cells. Wetland Science and Management. 2021, 17(04): 12-17.
11. Tang G., Shi Y., Liu S., et.al. Research progress of Constructed Wet-Microbial Fuel Cell Technology. Chemical Industry Environmental Protection. 2022, 42(05): 518-525.
12. Liu H., Hu T., Zeng G., et.al. Power generation characteristics of microbial fuel cells based on two phenolic organic compounds. Chinese Journal of Environmental Engineering. 2012, 6(01): 212-217.
13. Feng Y., Wang X., Wang H., et.al. Feasibility of combined electricity generation with cellulose-degrading bacteria and electrogenic bacteria using corn stalks as substrates. Acta Scientiae Circumologica Sinica. 2009, 29(11): 2295-2299.
14. Wu C., Zhang J., Wang Xiaoli., et.al. Study on the power generation characteristics of microbial fuel cells fueled by aniline and glucose. Acta Scientiae Circumologica Sinica. 2011, 31(06): 1227-1232.
15. Jiang S., Guan Y., Bai S., et.al. Study on the power generation performance of microbial fuel cells using phenol as fuel. Environmental Pollution and Control. 2013, 35(05): 19-23.
16. Luo H., Liu G., Zhang R. The power generation characteristics of microbial fuel cells using phenol as fuel. Acta Scientiae Circumologica Sinica. 2008(07): 1279-1283.
17. Jiang S., Guan Y., Bai S., et.al. Study on the power generation characteristics of microbial fuel cells with glucose as substrate. China Water & Wastewater. 2009, 25(23): 38-40.
18. Zhao L., Zou L., Wang X., et.al. An upflow single-chamber microbial fuel cell fueled by glucose. Journal of Daqing Petroleum Institute. 2010, 34(01): 76-79.
19. Xie Q. Leng G. Wang B., et.al. Study on response mechanism of MSBR-MFC integrated system with potassium ferricyanide as electron acceptor. Water Treatment Technology. 2011, 37(06): 120-125.
20. Zhao Y., Zhao Y., Guo L. Power generation performance of MFC fueled by pre-treated excess sludge and feasibility of discontinuous power supply. Environmental Science. 2016, 37(03): 1156-1162.
21. Zhang Y., Qiu Z., Zhao T., et.al. Construction of grid-like anodes and evaluation of their power generation performance in seabed sedimentary microbial fuel cells. Applied Chemical Industry. 2020, 49(S2): 155-159.
22. Li L., Wang H., Zhu D., et.al. Effects of mass transfer on the power generation performance and Atrazine degradation of soil microbial fuel cells. Journal of Southeast University (Natural Science Edition). 2018, 48(03): 455-462.
23. Guo S., Yang L., Gong Y., et.al. Enhancement of power generation characteristics of microbial fuel cells from excess sludge by low-temperature thermal oxidation. Environmental Chemistry. 2020, 39(05): 1330-1336.
24. Guo Z., Liu X., Huang T., et.al. Screening of light-dependent microorganisms and study on the electrical generation performance of MFC. Journal of Beijing University of Chemical Technology (Natural Science Edition). 2022, 49(06): 49-55.
25. Zhang H., Zhang T., Long X., et.al. Degradation characteristics of Cu-EDTA by coupling of photocatalysis and microbial fuel cells. Journal of Chemical Industry and Engineering. 2022, 73(05): 2149-2157.
26. Zhang Y., Li Y., Liu C., et.al. Study on the power generation characteristics of treated simulated wastewater using double-chamber microbial fuel cells. Environmental Science. 2012, 33(07): 2427-2431.
27. Xiong X., Wu X., Jia H., et.al. Treatment of Cr(Ⅵ) wastewater using Cu(Ⅱ) -enriched microbial fuel cells. Environmental Science. 2017, 38(10): 4262-4270.
28. He H., Feng Y., Li H., et.al. Construction of microbial fuel cells using Chlorella. Journal of Process Engineering. 2009, 9(01): 133-137.
29. Chen D. Research on electricity generation using microbial fuel cells with molasses wastewater as the substrate. Heilongjiang Agricultural Sciences. 2012(07): 116-118.
30. Gu D., Jia H., Wu Y., et.al. Simultaneous degradation of biogas slurry and triphenyltin chloride using microbial fuel cells. Journal of Chemical Industry and Technology. 2016, 67(05): 2056-2063.
31. Yin X., Liu W., Jiang L. Recovery of copper from copper-containing wastewater using microbial fuel cells. Environmental Engineering. 2014, 32(09): 152-157.
32. Lu N., Zhou S., Zhang J., et.al. Study on microbial fuel cells for generating electricity using corn soaking liquid. Environmental Science. 2009, 30(02): 563-567.
33. Cui Y., You H., Abuliti Abudula. Improving power generation performance of microbial fuel cells using bio-activated carbon. Progress in Chemical Industry. 2009, 28(11): 1917-1921.
34. Zhuang X., Chen W., Dai J., et.al. Study on microbial fuel cells constructed with algae and anaerobic sludge and their power generation and water purification performance. Municipal Technology. 2017, 35(06): 150-153.
35. Jin C., Yu H., Liu M., et.al. Study on the degradation of anthraquinone fuel active brilliant blue using persulfate cathodic microbial fuel cells. Journal of Ocean University of China (Natural Science Edition). 2015, 45(04): 85-94.
36. Shen J., Li J., Du Z., et.al. Study on the performance of single-chamber MFC in removing organic pollutants from coking wastewater. Water Treatment Technology. 2019, 45(04): 59-62.
37. Xie L., Chen L., Li R., et.al. Research on optimization of experimental conditions for simultaneous power generation in the treatment of simulated domestic wastewater by single-chamber air cathode fuel cell. Journal of Kunming Metallurgical College. 2020, 36(05): 87-90.
38. Hao X., Zhou X., Zhang J., et.al. Efficiency and mechanism of anaerobic fermentation sludge fuel cell in treating chromium-containing wastewater. Environmental Science in China. 2014, 34(10): 2581-2587.
39. Xu N., Liu X. Tandem treatment of beer wastewater by anaerobic fluidized bed microbial fuel cells. Shandong Chemical Industry. 2015, 44(09): 181-185.
40. Li D., Lou H. Preliminary study on Synergistic Denitrification by double-chamber microbial fuel cells. Journal of Dalian Minzu University. 2010, 12(03): 285.
41. Qian Y., Chen J., Zhao Z., et.al. Degradation of Rhodamine B dye wastewater by dual-chamber microbial fuel cells. Guangdong Chemical Industry. 2023, 50(07): 171-173.
42. Chen S., Wang J., Xia X., et.al. Dual-chamber microbial fuel cells simultaneously remove phenol and nitrate from wastewater. Chinese Journal of Environmental Engineering. 2012, 6(03): 891-895.
43. Sun C., Di X., Yu H., et.al. Study on electricity generation of silver-containing wastewater treated by double-chamber microbial fuel cells. Acta Scientiae Circumologica Sinica. 2015, 35(05): 1444-1448.
44. Sun C., Li L., Wang J., et.al. Study on the performance of double-chamber microbial fuel cells in treating organic wastewater and heavy metal wastewater. Water Treatment Technology. 2019, 45(08): 99-102.
45. Yang J., Wang J., Chen S., et.al. Treatment of nitrate wastewater by double-chamber microbial fuel cell. Chinese Journal of Environmental Engineering. 2013, 7(05): 1837-1842.
46. He Z., Zhang R., Long Q., et.al. Study on the removal of Cr (VI) from electrolytic manganese wastewater by double-chamber microbial fuel cell and its power generation performance. Proceedings of the Chinese Society for Nonferrous Metals. 2018, 28(09): 1937-1947.
47. Gao Y., Hai R., Wang X., et.al. Development of heavy metal toxicity sensor for dual-chamber microbial fuel cells. Chinese Journal of Environmental Engineering. 2017, 11(10): 5400-5408.
48. Lian J., Sun Y., Li H., et.al. Influencing Factors of Electricity Generation in Double-chamber sludge Microbial Fuel cells. Chinese Journal of Environmental Engineering. 2014, 8(10): 4515-4520.
49. Wang L., Cao X., Feng Y., et.al. Degradation of azo dyes and simultaneous power generation by double-layer anode microbial fuel cell system. Journal of Central South University (Natural Science Edition). 2019, 50(02): 264-271.
50. Na D., Zhang Y., Wang L., et.al. Study on the electricity generation of bipolar chamber microbial fuel cells in treating wastewater with different anode substrates. Water Treatment Technology. 2017, 43(02): 75-78.
51. Wen Q., Wu Y., Wang G., et.al. Microbial fuel cell for bipolar chamber combined treatment of beer wastewater. Acta Chimica Sinica of Higher Education Institutions. 2010, 31(06): 1231-1234.
52. Li X., Qu L. Research on the application of double-tank MFC reactor in domestic sewage treatment. Water Treatment Technology. 2013, 39(01): 109-113.
53. Geng Y., Lin X., Sun Y., et.al. Preparation of Bimetallic Conductive Metal-organic Framework Material Ni/Co-CAT for Electrocatalytic Oxygen Reduction[J]. Acta Chimica Sinica. 2022, 80(6): 748.
54. Li J., Zhang S. Basic research on denitrifying microbial fuel cells. China Environmental Science. 2012, 32(04): 617-622.
55. Jia J., Peng J., Wang Y., et.al. Study on nitrogen removal from low C/N wastewater by denitrifying microbial fuel cells. Environmental Pollution and Control. 2021, 43(08): 937-941.
56. Wen Q., Zhu N., Zhao L., et.al. Upflow air cathode biofuel cell for simultaneous wastewater treatment and production capacity. Water Treatment Technology. 2009, 35(09): 26-29.
57. Cai J., Liu S., Wu Y., et.al. Study on electrochemical characteristics of fuel cells for simultaneous nitrogen and sulfur removal. Journal of Zhejiang University (Science Edition). 2022, 49(01): 105-111.
58. Wang C., Zhang Y., Wu S., et.al. Effects of solid Content and Electrode Spacing on the Electricity Generation Performance of Cow Dung Fermentation. Chinese Journal of Environmental Engineering. 2016, 10(01): 485-489.u\
59. Guo H., Zhou L., Tang S., et.al. Microbial fuel cell treatment technology for oily sludge. Journal of Yangtze University (Natural Science Edition). 2020, 17(03): 61-65.
60. Guo H., Wei L., Geng X., et.al. Electricity generation performance of oily sludge bioelectrochemical system and characteristics of anode membrane flora variation. Science, Technology and Engineering. 2020, 20(19): 7952-7957.
61. Zhang C., Xie J., Jia H. Study on the power generation characteristics of quinoline as a single fuel in filled MFC. Environmental Science and Technology. 2014, 37(10): 31-36.
62. Wang H., Li L., Cao X., et.al. Power generation performance and microbial community structure analysis of soil microbial fuel cells under different conditions. Journal of Southeast University (Natural Science Edition). 2017, 47(06): 1141-1147.
63. Zhang L., Dou C., Shi H., et.al. Experimental study on nitrification process at the bio-cathode of microbial fuel cells. Environmental Pollution and Control. 2012, 34(09): 8-12.
64. Sun Y., Lian J., Lyu G., et.al. Constructing microbial fuel cells in oxidation ditches. Chemical Industry Environmental Protection. 2012, 32(02): 105-108.
65. Gao X., Guo C., Shi Y., et.al. Power generation performance of microbial fuel cells from landfill leachate to sedimentary sludge. Chinese Journal of Environmental Engineering. 2016, 10(11): 6519-6523.
66. Tang C. Research on the Treatment of Malachite Green Simulated Wastewater and Synchronous Production Capacity Based on Air Cathode Microbial Fuel Cells. Clean World. 2023, 39(03): 60-63.
67. Jin C., Wang P., Yu H., et.al. Microbial fuel cells based on air cathode for treating chromium-containing (Ⅵ) wastewater. Journal of Ocean University of China (Natural Science Edition). 2015, 45(05): 69-74.
68. Wang S., Liu B., Tan G., et.al. Investigation on influencing factors of autotrophic denitrification efficiency of biological cathodes based on iron anodes. Acta Scientiae Circumologica Sinica. 2022, 42(08): 137-145.
69. Cui L., Zuo J., Fan M. Microbial fuel cells for treating urban sewage and generating electricity. Biogas in China. 2006(04): 3-5.
70. Xie M., Xu L., Cheng L. Study on Performance of microbial fuel cells using treated aged landfill leachate as cathode liquid. Acta Energiae Sinica. 2018, 39(09): 2641-2647.
71. Wei D., Zhou X., Zhang J., et.al. Study on the migration and removal of copper and lead by microbial fuel cells in composite contaminated soil. Energy and Environment. 2023(01): 12-16.
72. Wang L., Li X., Wang L. Wastewater treatment and simultaneous power generation performance of microbial fuel cells with composite biological cathode. Research of Environmental Sciences. 2017, 30(07): 1098-1104.
73. Li H. Study on the power generation of microbial fuel cells enhanced by composite carbon nanoanodes. Journal of South China Normal University (Natural Science Edition). 2016, 48(04): 45-49.
74. Zhu R., Ren Y., Li X., et.al. Effects of external resistance on the operational characteristics of sedimentary microbial fuel cells (SMFC) in natural water bodies. Environmental Chemistry. 2014, 33(05): 794-798.
75. Xie S., Liang P., Li L., et.al. Study on the combined process of aerobic/anoxic bio-cathode-type microbial fuel cells for treating nitrogen-containing wastewater. Guangdong Chemical Industry. 2011, 38(06): 126-127.
76. Li L., Guan Y., Yang M. Research on power generation performance and mechanism analysis of Chlorella microbial fuel cells. Shandong Chemical Industry. 2016, 45(23): 26-28.
77. Liu R. Research on nitrogen removal in microoxygen bio-cathode microbial fuel cells. Chemical Engineering Design Communications. 2021, 47(05): 114-115.
78. Yang S., Liu Z., Hou J., et.al. Preparation and performance of MnO_2_/S-AC foam nickel air cathode for microbial fuel cells. Journal of Chemical Industry and Technology. 2015, 66(S1): 202-208.
79. Tang Y. Experimental study on different ionic strengths of microbial fuel cells. Journal of Beijing Union University (Natural Science Edition). 2015, 29(02): 67-73.
80. Tang Y. Research on the Electricity Generation Performance of Different Anode Substrates in Microbial Fuel Cells. Journal of Jianghan University (Natural Science Edition). 2018, 46(03): 210-215.
81. Li Y., Sun Y., Kong X., et.al. Isolation and characterization of electrogenic bacteria Citrobacter freundii in microbial fuel cells. Journal of Solar Energy. 2012, 33(11): 1968-1972.
82. Fei J., Teng Y., Xiong L., et.al. Study on the power generation performance of Pseudomonas F026 in microbial fuel cells. Journal of Microbiology. 2015, 35(01): 35-39.
83. Zhu J., Su W. Study on the complex flow mechanism of electrolyte in porous electrodes in microbial fuel cells. Energy and Environment. 2014(03): 7-8.
84. Huang M., Wang J., Zhu C. Discussion on salt bridge connection in microbial fuel cells. Journal of Hefei University of Technology (Natural Science Edition). 2008(10): 1574-1576.
85. Li J., Li S., Liu C. Experimental study on power generation performance of microbial fuel cells. Renewable Energy. 2014, 32(10): 1564-1568.
86. Fu J., Qi T., Cai X., et.al. Study on power generation of microbial fuel cells and microbial diversity analysis. Chinese Journal of Applied and Environmental Biology. 2009, 15(04): 568-573.
87. Chen L., Hu Y., Wang Z. Discussion on the application of microbial fuel cells in Marine domestic Sewage treatment. Shipbuilding Technology. 2016(01): 1-3.
88. Zhang X., Zhao S., Li Y, et.al. Study on the treatment of CTMP pulp wastewater by microbial fuel cells. China Paper. 2011, 30(09): 6-10.
89. Pan W., Ji Z., W, et.al. Power generation performance and degradation process of microbial fuel cells in treating azo saline Wastewater. Progress in Chemical Industry. 2022, 41(06): 3306-3313.
90. Peng J., Guo Y. Research on the treatment of azo dyes by microbial fuel cells. Environmental Science and Management. 2014, 39(07): 45-49.
91. Zhou Y. Experimental study on the treatment of pig wastewater by microbial fuel cells. Energy and Environment. 2017(02): 19-20.
92. Zhao Q., Jiang J., Wang K., et.al. Microbial fuel cell treatment of excess sludge and synchronous power generation performance. Journal of Harbin Engineering University. 2010, 31(06): 780-785.
93. Chen W., Liu Z, Jiang K., et.al. Study on the treatment of sodium citrate wastewater by microbial fuel cells. Journal of Chemical Industry and Technology. 2019, 70(S2): 322-328.
94. Zhao L., Kong F., Wang X., et.al. Microbial fuel cells for treating chromium-containing wastewater and generating electricity simultaneously. Modern Chemical Industry. 2009, 29(11): 37-39.
95. Tian Y., Yang J., Cheng S., et.al. Research on microbial fuel cells for wastewater power generation and their driving monitoring system. Journal of Zhengzhou University (Engineering and Technology Edition). 2018, 39(01): 90-96.
96. Fan X., Cheng Y., Lu K. Experimental study on microbial fuel cells for treating domestic sewage. Environmental Science and Technology. 2015, 38(11): 211-215.
97. Liu Y Wang Z., Liu J., et.al. Microbial fuel cell treatment of direct red simulated wastewater. Journal of Qingdao University of Science and Technology (Natural Science Edition). 2016, 37(02): 180-184.
98. Zhan H., Su X., Sha J., et.al. Study on the characteristics of microbial fuel cells in treating casings wastewater. Journal of Harbin University of Commerce (Natural Science Edition). 2015, 31(06): 682-685.
99. Tong W., Zhang S., Wang M. Microbial fuel cells for treating ship bilge water. Journal of Shandong Jiaotong University. 2020, 28(03): 77-82.
100. Ding W., Wang J., Lyu J., et.al. Microbial fuel cell treatment of phenol wastewater. Journal of Hefei University of Technology (Natural Science Edition). 2010, 33(01): 94-96.
101. Wang J., Xia X., Ding W. Study on operating conditions of microbial fuel cells for treating phenol wastewater. Acta Scientiae Circumstantica Sinica. 2010, 30(04): 735-741.
102. Zhang J., Zhao Q., Yuan Y., et.al. Microbial fuel cell method for determining BOD in domestic sewage. Journal of Harbin Institute of Technology. 2010, 42(11): 1788-1792.
103. Liang F., Feng W., Su Z., et.al. Microbial fuel cell electrodes for the treatment of OCC wastewater. Journal of Chinese Paper. 2016, 31(02): 24-28.
104. Zheng W., Tang C., Huang M., et.al. Performance comparison and membrane fouling analysis of algal cathodes in microbial fuel cells. Chinese Journal of Environmental Engineering. 2016, 10(03): 1211-1216.
105. Lin Y., Liu L., Yang F. Water treatment characteristics and electricity generation behavior of modified stainless steel cathode film coupled bioelectrochemical system. Membrane Science and Technology. 2015, 35(05): 30-34.
106. Liu W., Yin X., Lu J., et.al. Copper recovery and electricity generation performance from copper-containing wastewater treated by membrane-free microbial fuel cells. Proceedings of the Chinese Society for Nonferrous Metals. 2017, 27(03): 648-654.
107. Wang J., Shen Q., Yang Y. Study on the remediation and mechanism of Cr (VI) contaminated wetland soil by plant-microbial fuel cells. Acta Scientiae Circumologica Sinica. 2019, 39(02): 518-526.
108. Liu Z., Li X., Fang L., et.al. Research on the Operating Characteristics of Microbial Fuel Cells using Sludge as Fuel. China Environmental Science. 2012, 32(02): 268-273.
109. Hu J., Dai J., Sun Z., et.al. Research on sediment microbial fuel cells in sludge pollution removal. Waterway Port. 2020, 41(06): 725-730.
110. Song L., Zhang D., Bai R., et.al. Study on the performance of microbial fuel cells catalyzed by a mixture of activated carbon and TiO_2_ with nickel-based bio-cathode. Renewable Energy. 2015, 33(04): 612-617.
111. Liu R., Wang X., Hai R., et.al. Activated carbon optimizes bio-cathode to enhance power generation performance of microbial fuel cells. Acta Scientiae Circumologica Sinica. 2015, 35(07): 2059-2063.
112. Liu Z., Zhou Y., Hou J., et.al. Study on anodes of microbial fuel cells modified by activated carbon. Water Treatment Technology. 2014, 40(04): 16-18.
113. Guo J., Pan B., Ye Y., et.al. Study on amplification and series-parallel combination of activated carbon air cathode microbial fuel cells. Modern Chemical Industry. 2013, 33(10): 99-103.
114. Wang F., Wang T., Li J., et.al. Research on Thermal cracking Treatment of Duckweed and its Electricity generation Performance in Microbial Fuel Cells. Journal of Shanghai Normal University (Natural Science Edition). 2021, 50(04): 478-486.
115. Zhang Y., Fu Y., Lu Z., et.al. Electrocatalytic degradation of organic carbon by subsea microbial fuel cells. Chemical Engineering. 2013, 41(05): 10-13.
116. Li K., Fu Y., Xu Q., et.al. Study on anode manganese salt modification and power generation performance of subsea microbial fuel cells. Materials Development and Application. 2011, 26(03): 46-49.
117. Zhao L., Song J., Yu J., et.al. Preliminary study on the power generation performance of hybrid microbiota and single-strain microbial fuel cells. Chemical and Biological Engineering. 2013, 30(01): 64-67.
118. Zhang T., Zhang L., Gao P., et.al. Power generation mechanism and characteristics of hybrid microbiota biofuel cells. Chinese Journal of Applied and Environmental Biology. 2012, 18(03): 465-470.
119. Yuan Y., Li., Fu S., et.al. Study on power generation characteristics of microbial fuel cell chips under mixed bacterial inoculation conditions. Acta Scientiae Circumologica Sinica. 2014, 34(05): 1186-1191.
120. Li R., Ren Y., Wang C. Study on the effects of temperature and stirring on microbial fuel cell performance. Gansu Science and Technology. 2015, 31(01): 43-46.
121. Zhu X., Liao C., Han Y., et.al. Effects of temperature on electrochemical performance of electroautotrophic oxygen reduction biological cathodes. Acta Scientiae Circumologica Sinica. 2022, 42(05): 208-216.
122. Wu D., Ding Q., Wang X., et.al. Study on the electrical generation performance of sugar beet residue in microbial fuel cells. Journal of Harbin University of Commerce (Natural Science Edition). 2013, 29(02): 153-155.
123. Yang G., Jiang J., Wang K., et.al. The efficiency of bio-generated constructed wetland system in treating domestic sewage. Journal of Zhejiang University (Engineering and Technology Edition). 2015, 49(06): 1186-1192.
124. Huang G., Jiang J., Zhao Q., et.al. biological electricity generation accelerates anaerobic composting sludge degradation and electricity generation performance. Journal of Zhejiang University (Engineering Science). 2013, 47(05): 883-888.
125. Wang S., Jiang J., Zhao Q., et.al. Bioelectricity generation accelerates organic matter degradation in sludge wetlands. Journal of Harbin Institute of Technology. 2016, 48(08): 48-54.
126. Zheng B., Feng H., Wang Y., et.al. Study on power generation characteristics and COD removal effect of microbial fuel cells by bio-enhanced process. Jiangsu Agricultural Sciences. 2012, 40(10): 339-341.
127. Wang D., Song T., Wu X., et.al. Research progress on bioelectrochemical removal of pollutants. Modern Chemical Industry. 2014, 34(03): 29-33.
128. Wang J., Huang W., Wang X., et.al. Application of biofilm electrodes in microbial fuel cells fueled by phenol. Acta Chimica Sinica of Higher Education Institutions. 2013, 34(04): 975-979.
129. Zhang J., Zhao Q., You S., et.al. Electricity generation characteristics of different cathode materials in bio-cathode microbial fuel cells. Acta Chimica Sinica of Higher Education Institutions. 2010, 31(01): 162-166.
130. Fan L., Xu D. Influence of electrochemical modified anode on MFC performance. Journal of Fuel Chemistry and Technology. 2016, 44(05): 628-633.
131. Zhao S., Liu S., Xue M., et.al. Study on the effect of electrochemically polymerized polyaniline modified electrodes on the power generation performance of microbial fuel cells. Journal of Henan University (Natural Science Edition). 2020, 50(01): 87-93.
132. Liu Y., Sun W., Gong L. The effect of electron acceptors on the power generation performance of microbial fuel cells. Environmental Pollution and Control. 2016, 38(11): 84-89.
133. Lu Y., Liu J., Wang X., et.al. Effects of electrical conductivity on the performance of anaerobic acid production, forward osmosis and microbial fuel cell coupling process. Environmental Science. 2018, 39(07): 3240-3246.
134. Yin X., Liu W. Electrode effects on the power generation performance of microbial fuel cells in treating organic wastewater and copper-containing heavy metal wastewater simultaneously. Advances in Chemical Industry. 2015, 34(04): 1152-1158.
135. Qin Y., Lin X., Zheng L., et.al. Research progress on electrode-modified enhanced microbial fuel cell power generation for simultaneous degradation of organic pollutants. Fine Chemicals. 2021, 38(09): 1737-1745.
136. Xie M., Xu L., Hu J. Influence of electrode materials on the performance of microbial fuel cells in treating aged landfill leachate. Acta Energiae Sinica. 2019, 40(02): 319-325.
137. Wang M., Zhao Y., Lu S. Analysis of the dynamic characteristics of microbial community structure during the isolation of electrode active bacteria. Environmental Science. 2014, 35(10): 3940-3946.
138. Liu T., Xu D., Wang L., et.al. Effect of electrode spacing on Zn and Ni in sludge treated by CW-MFC and its power generation performance. Advances in Chemical Industry. 2021, 40(07): 4074-4082.
139. Bian X., Yu J., Wang Y., et.al. Performance of electrolysis - microbial fuel cell coupling system in treating dye wastewater. Industrial Water Treatment. 2020, 40(10): 59-62.
140. Xu Y., Song T., Ye Y., et.al. Preparation and optimization of cathode for direct microbial fuel cells. Journal of Process Engineering. 2008(05): 998-1002.
141. Cai W., Chen S., Wen Q. Construction and power generation performance of direct air cathode biofuel cells. Journal of Luoyang Institute of Science and Technology (Natural Science Edition). 2010, 20(02): 8-11.
142. Liu Ci., Sun C., Gao Y., et.al. Performance study of direct aluminum-sodium persulfate microfluidic fuel cells. Journal of Henan University of Science and Technology (Natural Science Edition). 2023, 44(02): 25-31.
143. Yang X., Wang K., Feng C., et.al. Graphene-oxide aerogel-modified metal anodes for enhancing the power generation performance of microbial fuel cells. Chinese Journal of Environmental Engineering. 2017, 11(04): 2598-2606.
144. Zheng Z., Zhou S., Li C., et.al. Study on silane coupling agent modified MFC anodes and their battery performance. China Water & Wastewater. 2018, 34(13): 125-129.
145. Tang Y., Sun Y., Yu J., et.al. Properties of nitric acid chemically modified carbon felt air cathode. China Water & Wastewater. 2014, 30(05): 10-14.
146. Wang H., Liu L., Hu H. Electricity generation characteristics of microbial fuel cells with alkali-treated sludge as substrate. China Water & Wastewater. 2012, 28(07): 5-8.
147. Liu S., Chen L., Huang M., et.al. Power generation performance of microbial fuel cells using two bio-cathode materials, carbon felt and carbon cloth. Chinese Journal of Environmental Engineering. 2014, 8(10): 4540-4544.
148. Li M., Cheng S. Research on process optimization of air-cathode microbial fuel cell stack. Modern Chemical Industry. 2020, 40(08): 185-189.
149. Qi F., Cai C., Jiang L., et.al. Study on the treatment of simulated acidic mine water by air cathode microbial fuel cell. Journal of Anhui Polytechnic University. 2014, 29(03): 1-4.
150. Qiang L., Yuan L., Ding Q. Study on the power generation characteristics of air cathode microbial fuel cells for treating domestic sewage. Water Treatment Technology. 2011, 37(01): 76-79.
151. Wen Q., Liu Z., Chen Y., et.al. Electrochemical performance of air cathode biofuel cells. Acta Physico-Chimica Sinica. 2008(06): 1063-1067.
152. Zhang J., Zhao S., Zhou Y., et.al. Effects of concentrated neutral red modified electrodes on nitrogen removal and power generation performance of microbial fuel cells (MFC). Journal of Zhejiang University (Science). 2019, 46(05): 589-599.
153. Sun Y., Liu W. Application of polymer-modified anodes for power generation and cobalt leaching in microbial fuel cells. Chinese Journal of Nonferrous Metals. 2020, 30(05): 1084-1090.
154. Guo X., Wang Q., Cheng X. Effects of polymer-modified anodes on electrochemical performance and cobalt leaching of PEMFC. Powder Metallurgy Industry. 2022, 32(05): 85-89.
155. Jia Y., Qi Q., Wang X. Preparation of polypyrrole/carbon nanotube membrane anodes and their application in anaerobic fluidized bed microbial fuel cells. Journal of Qingdao University of Science and Technology (Natural Science Edition). 2017, 38(02): 42-47.
156. Zhao T., Qiu Z., Zheng J., et.al. Research on polypyrrolid-modified carbon brush electrode in microbial fuel cells. Modern Chemical Industry. 2021, 41(10): 186-190.
157. Li Y., Li H. Polyaniline/FE-N-reduced graphene modified conductive film-coupled membrane bioreactor/Microbial fuel cell Wastewater treatment study. Journal of Southwest Minzu University (Natural Science Edition). 2018, 44(03): 282-286.
158. Zang C., Huang C., Yin J., et.al. Effects of polyaniline/carbon nanotube modified cathode on power generation performance of microbial fuel cells. Shandong Industrial Technology. 2018(20): 15-16.
159. Ren Y., Pan D., Li X., et.al. Effects of polyaniline-modified cathode on power generation performance of sedimentary microbial fuel cells. Journal of Jiangnan University (Natural Science Edition). 2013, 12(06): 714-718.
160. Wang Q., Liu P., Weng X., et.al. Nitrogen removal and electricity generation of the bio-cathode of the microbial fuel cell of Streptococcus denitrogenated YF1. Chinese Journal of Environmental Engineering. 2014, 8(08): 3277-3282.
161. Ge A., Qin W., Jin Q. Study on the power generation performance of self-made coated three-dimensional anode MFC. Power Supply Technology. 2016, 40(01): 94-96.
162. Ren Y., Fu F., Li X., et.al. Effects of sodium anthraquinone-2, 6-disulfonic acid-doped polypyrrole modified cathode on power generation performance of sedimentary microbial fuel cells. Environmental Chemistry. 2013, 32(10): 1851-1855.
163. Cui Y., Su W., Gao P., et.al. Degradation of coupled azo dyes in reductive sulfide microbial fuel cells. Chinese Journal of Applied and Environmental Biology. 2012, 18(06): 978-982.
164. Zhang W., Wu M., Xu X., et.al. Study on enhancing the efficiency of sedimentary microbial fuel cells with reduced iron powder. Water Treatment Technology. 2021, 47(05): 102-105.
165. Ding W., Aruna., Fu Z., et.al. Effects of heavy metal ions on power generation performance of microbial fuel cells. Environmental Engineering. 2016, 34(07): 61-65.
166. Zhu M., Liu W. Electrical generation performance of MFC with copper ion as electron acceptor and wastewater treatment. Industrial Water Treatment. 2017, 37(12): 64-68.
167. Mu S., Li X., Ren Y., et.al. Study on the effect of copper ions on the power output of dual-chamber microbial fuel cells. Environmental Science. 2014, 35(07): 2791-2797.
168. Niu Y., Chen Z., Zhao S., et.al. Isolation and screening of chromium-reducing bacteria and their application in biological cathodes of microbial fuel cells. Chinese Journal of Microbiology. 2017, 44(07): 1631-1638.
169. Ma Z., Niu Y., Zhao J., et.al. Effects of anode modification on microbial fuel cell performance. Science, Technology and Engineering. 2021, 21(18): 7820-7826.
170. Yin Y., Huang G., Tong Y., et.al. Effects of anode loading steady magnetic field on microbial fuel cell performance. Chemical World. 2014, 55(02): 92-97.
171. Zhong D., Chen Y., Liang P., et.al. Effects of anode thickness on power generation performance of packed microbial fuel cells. China Water & Wastewater. 2009, 25(07): 9-12.
172. Xu Y., Xu L., Hu J. Effects of anode modification on performance of single-cell microbial fuel cells. Journal of Fuel Chemistry and Technology. 2018, 46(05): 600-606.
173. Sun Y., Liu W., Xu J. Effects of anodic modification on power generation and wastewater treatment in microbial fuel cells. Water Treatment Technology. 2020, 46(10): 39-43.
174. Wang M., Liu T., Zhang X., et.al. Effects of anodic modification on the performance of microbial fuel cells in treating straw hydrolyzed products. Journal of Fuel Chemistry. 2017, 45(09): 1146-1152.
175. Ding W., Yu L., Chen J., et.al. Effects of anode materials on performance and organic wastewater treatment of 6L microbial fuel cells. Environmental Science. 2017, 38(05): 1911-1917.
176. Xiang L., Wang X., Hai R., et.al. Research on the Influence of Anode Materials on the Power Generation Performance of Bio-cathode Type Microbial Fuel Cells. Water Treatment Technology. 2015, 41(07): 45-48.
177. Guo J., Kang X., Gao J., et.al. Effects of anode carbon materials on the startup of single-chamber air cathode microbial fuel cells. Journal of Dalian Polytechnic University. 2020, 39(01): 36-40.
178. Zhong L., Ma Y., Li T., et.al. Effects of anode carbon paper overlay on the power generation performance of microbial fuel cells. New Materials for Chemical Industry. 2015, 43(03): 126-129.
179. Jiang Y., Xu Y., Yang Q., et.al. Effects of cathode catalysts and different substrates on microbial fuel cells. Chemical Industry Environmental Protection. 2013, 33(05): 431-436.
180. Huang J., Yang P., Guo Y., et.al. Effects of cathode liquid and substrate concentration on concurrent wastewater treatment and power generation performance of AFB-MFC. Chinese Journal of Environmental Engineering. 2012, 6(02): 462-466.
181. Lu X., Hou B., Wang H., et.al. Study on the effects of acclimation methods on the treatment of coking wastewater and electricity generation by microbial fuel cells. Science, Technology and Engineering. 2017, 17(07): 42-45.
182. Shen J., Hu C., Xin Y., et.al. Effects of potassium permanganate on long-term power generation performance of cow dung microbial fuel cells. China Environmental Science. 2018, 38(05): 1712-1718.
183. Wen Q., Liu Z., Chen Y., et.al. Potassium permanganate for Cathode Electron Acceptors in Biofuel cells. Power Supply Technology. 2008(09): 584-587.
184. Yin Y., Yuan L., Niu Y. Relationship between liquid volume variation in two chambers of DCMFC and power generation performance. Journal of Chemical Industry and Technology. 2018, 69(08): 3605-3610.
185. Huo Q., Huang R., Qi W., et.al. Preparation of GO/PEDOT composite modified anode and its application in MFC. Journal of Chemical Industry and Technology. 2016, 67(10): 4406-4412.
186. Feng J., Yao H., Cai C., et.al. Study on CuO-enhanced MFC-activated persulfate degradation of azo dye wastewater and simultaneous electricity generation. Acta Scientiae Environmentalis. 2019, 39(04): 1157-1165.

Appendix Ⅱ: References used in the comparison with this study

1. Agrahari, R., Bayar, B., Abubackar, H.N., Giri, B.S., Rene, E.R. & Rani, R. 2022. Advances in the development of electrode materials for improving the reactor kinetics in microbial fuel cells. Chemosphere, 290, 133184.
2. Amin, M.M., Arvin, A., Feizi, A., Dehdashti, B. & Torkian, A. 2022. Meta-analysis of bioenergy recovery and anaerobic digestion in integrated systems of anaerobic digestion and microbial electrolysis cell. Biochemical Engineering Journal, 178, 108301.
3. Bird, H., Heidrich, E.S., Leicester, D.D. & Theodosiou, P. 2022. Pilot-scale Microbial Fuel Cells (MFCs): A meta-analysis study to inform full-scale design principles for optimum wastewater treatment. Journal of Cleaner Production, 346, 131227.
4. Dowdy, F.R., Kawakita, R., Lange, M. & Simmons, C.W. 2018. Meta-analysis of Microbial Fuel Cells Using Waste Substrates. Applied Biochemistry and Biotechnology, 185, 221-232.
5. Fouchecour, F., Larzilliere, V., Bouchez, T. & Moscoviz, R. 2022. Systematic and quantitative analysis of two decades of anodic wastewater treatment in bioelectrochemical reactors. Water Research, 214, 118142.
6. Hindatu, Y., Annuar, M.S.M. & Gumel, A.M. 2017. Mini-review: Anode modification for improved performance of microbial fuel cell. Renewable & Sustainable Energy Reviews, 73, 236-248.
7. Kumar, R., Singh, L., Zularisam, A.W. & Hai, F.I. 2018. Microbial fuel cell is emerging as a versatile technology: a review on its possible applications, challenges and strategies to improve the performances. International Journal of Energy Research, 42, 369-394.
8. Kusmayadi, A., Leong, Y.K., Yen, H.W., Huang, C.Y., Dong, C.D. & Chang, J.S. 2020. Microalgae-microbial fuel cell (mMFC): an integrated process for electricity generation, wastewater treatment, CO2 sequestration and biomass production. International Journal of Energy Research, 44, 9254-9265.
9. Mohyudin, S., Farooq, R., Jubeen, F., Rasheed, T., Fatima, M. & Sher, F. 2022. Microbial fuel cells a state-of-the-art technology for wastewater treatment and bioelectricity generation. Environmental Research, 204, 112387.
10. Nosek, D., Jachimowicz, P. & Cydzik-Kwiatkowska, A. 2020. Anode Modification as an Alternative Approach to Improve Electricity Generation in Microbial Fuel Cells. Energies, 13, 6596.
11. Selvasembian, R., Mal, J., Rani, R., Sinha, R., Agrahari, R., Joshua, I., Santhiagu, A. & Pradhan, N. 2022. Recent progress in microbial fuel cells for industrial effluent treatment and energy generation: Fundamentals to scale-up application and challenges. Bioresource Technology, 346, 126462.
12. Uddin, M.J., Jeong, Y.K. & Lee, W. 2021. Microbial fuel cells for bioelectricity generation through reduction of hexavalent chromium in wastewater: A review. International Journal of Hydrogen Energy, 46, 11458-11481.
13. Zafar, H., Ishaq, S., Peleato, N. & Roberts, D. 2022. Meta-analysis of operational performance and response metrics of microbial fuel cells (MFCs) fed with complex food waste. Journal of Environmental Management, 315, 115152.
